# Lysine lactylation‐mediated post‐translational modification: Molecular mechanisms and therapeutic target exploration in tumour drug resistance

**DOI:** 10.1002/ctm2.70714

**Published:** 2026-06-17

**Authors:** Juan Li, Yan Shang, Haojie Wu, Shenglong Yang, Maiqun Zuo, Hailong Zhao

**Affiliations:** ^1^ Department of Pathophysiology Zunyi Medical University Zunyi Guizhou China; ^2^ Department of Gastroenterology Guizhou Sinan County People's Hospital Tongren Guizhou China; ^3^ School of Preclinical Medicine of Zunyi Medical University Guizhou China

**Keywords:** DNA damage repair, immune evasion, lysine lactylation (Kla), post‐translational modification, tumour drug resistance, Warburg effect

## Abstract

**Background:**

Tumour drug resistance remains a major hurdle in cancer treatment, with post‐translational modifications (PTMs) playing a pivotal role in this process. Lysine lactylation (Kla), a newly identified PTM, is tightly associated with the Warburg effect, where tumour cells favour glycolysis even under aerobic conditions, resulting in excessive lactate accumulation. As a key substrate for Kla, this surplus lactate leads to markedly elevated Kla levels in tumours.

**Content and Focus:**

This review systematically outlines the molecular mechanisms of Kla, including its biochemical definition, enzymatic regulation, metabolic cross‐talk and feedback mechanisms within tumour reprogramming. Furthermore, we comprehensively discuss how Kla promotes tumour drug resistance through diverse mechanisms: activating DNA damage repair pathways, mediating epigenetic regulation and gene expression reprogramming, remodelling the tumour microenvironment (TME) to facilitate immune evasion and disrupting the balance between cell survival and apoptosis. Additionally, we analyse Kla's context‐specific characteristics and mechanisms in drug resistance across distinct cancer types, such as breast, colorectal, lung and pancreatic cancer. Notably, we further expand the discussion to the spatial and clonal heterogeneity of Kla within the same tumour, including the divergent Kla profiles between hypoxic tumour cores and oxygen‐rich tumour peripheries, the distinct Kla status of circulating tumour cells compared with primary tumour sites, and the specific Kla signatures that distinguish drug‐resistant clones from drug‐sensitive counterparts, key biological features that are critical for understanding Kla‐driven drug resistance but have been largely understudied. Finally, we explore potential Kla‐targeted therapeutic strategies, including inhibition of key lactate‐metabolising enzymes, modulation of lactyltransferase activity and combination therapies, while discussing current challenges and future directions.

**Conclusions:**

By unravelling these Kla‐driven mechanisms, this review provides a novel metabolic–epigenetic axis for overcoming cancer therapeutic resistance. Kla serves as a metabolic–epigenetic switch governed by writer–eraser–reader regulatory systems, directly connecting the Warburg effect to diverse forms of tumour drug resistance. Pharmacological modulation of Kla offers an innovative precision therapeutic approach to reverse drug resistance and enhance clinical prognosis across various malignancies.

**Key points:**

- Lysine lactylation (Kla) functions as a metabolic‐epigenetic switch that directly links the Warburg effect to tumour drug resistance via the writer‐eraser‐reader regulatory framework
- Kla promotes drug resistance through four interconnected mechanisms: DNA damage repair activation, epigenetic reprogramming, immune microenvironment remodelling, and anti‐apoptotic pathway engagement.
- Intratumoural Kla exhibits profound spatial and clonal heterogeneity, with distinct Kla profiles in hypoxic tumour cores, oxygen‐rich peripheries, circulating tumour cells, and drug‐resistant clones.
- Kla‐targeted therapeutic strategies—including inhibition of lactate‐metabolising enzymes, modulation of lactyltransferase/delactylase activity, and combination therapies—offer a novel precision approach to reverse drug resistance.

## Graphical abstract

Lysine lactylation (Kla) serves as a metabolic–epigenetic switch governed by writer–eraser–reader regulatory systems, directly connecting the Warburg effect to diverse forms of tumour drug resistance.

Intratumoural lactylation critically contributes to treatment failure in chemotherapy, targeted therapy and immunotherapy.

Pharmacological modulation of lysine lactylation (Kla) offers an innovative precision therapeutic approach to reverse drug resistance and enhance clinical prognosis across various malignancies.

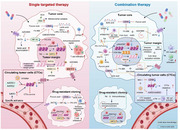

## 1 INTRODUCTION

Tumour drug resistance poses a serious threat to patient survival and remains a leading cause of cancer treatment failure.^1^, ^2^ Clinical studies indicate that approximately 70% of advanced cancer patients develop resistance during therapy, leading to disease relapse, metastasis and a significant decline in 5‐year survival rates.^3^ Post‐translational modifications (PTMs) play a critical role in mediating tumour drug resistance, whose dysregulation forms a complex regulatory network that synergistically propels the onset and progression of treatment resistance.^4^

Lysine lactylation (Kla) is a recently discovered PTM of proteins. Traditionally, lactate was considered merely as a metabolic byproduct of anaerobic glycolysis with no recognised biological activity beyond this role. However, a landmark study by Zhang and colleagues uncovered that lactate can act as a signalling molecule by covalently modifying lysine residues on target proteins, thereby exerting regulatory control over key biological processes.^5^ Subsequent studies have further established Kla as a widespread regulatory mechanism in cancer, linking high glycolytic flux to transcriptional reprogramming and therapeutic resistance.^5^, ^6^ This discovery carries profound implications for cancer biology, given the distinctive metabolic phenotype of tumour cells characterised by the Warburg effect. Even under aerobic conditions, cancer cells exhibit a preferential reliance on glycolysis, which results in extensive lactate generation and subsequent elevation of lactate concentrations both intracellularly and within the tumour microenvironment (TME).^6^ This lactate‐rich environment provides abundant substrate for Kla, ultimately leading to a marked up‐regulation of Kla levels in tumour cells.^7^ Importantly, recent lactylomic studies have revealed hundreds of Kla sites on both histone and non‐histone proteins, implicating Kla in diverse cellular processes beyond metabolism, including immune regulation and DNA damage response.^8^, ^9^ Notably, Kla is intricately linked to tumour metabolic reprogramming. It can modify both glycolytic enzymes and mitochondrial‐associated proteins, thereby steering metabolic pathway selection and driving cancer cells towards a glycolysis‐dependent phenotype that sustains their survival advantage.^8^ Critically, emerging evidence underscores the pivotal role of Kla in directly subverting apoptotic cell death, a key mechanism of cytotoxic therapies. Kla has been shown to modify and stabilise anti‐apoptotic proteins such as BCL‐2 and MCL‐1, while concurrently inhibiting the activity of pro‐apoptotic factors, thereby establishing a formidable barrier against therapy‐induced apoptosis.^10^ The role of Kla in promoting tumour drug resistance has gained increasing attention. Recent evidence demonstrates that Kla modulates the expression and function of key resistance effectors, such as drug efflux pumps, anti‐apoptotic proteins and immune checkpoint molecules, thereby enabling cancer cells to evade the cytotoxic effects of chemotherapeutic agents, targeted therapies and immunotherapies.^11^, ^12^

Tumours are not homogeneous cellular entities but exhibit extensive spatial and clonal heterogeneity, which is a core driver of therapeutic failure and clinical relapse.^13^ Kla, as a metabolism‐dependent PTM, is inherently subject to the spatial metabolic differences within the TME and the genetic/epigenetic divergence of tumour clonal populations.^2^ The hypoxic tumour core, characterised by severe oxygen deprivation and robust glycolysis, accumulates high levels of lactate and exhibits distinct Kla profiles compared with the relatively oxygen‐rich tumour periphery with moderate glycolytic activity.^14^ Additionally, circulating tumour cells (CTCs), which detach from the primary tumour and enter the systemic circulation to mediate distant metastasis, encounter a drastically different microenvironmental niche (e.g., low glucose, dynamic oxygen tension) and display Kla status that is distinct from primary tumour cells.^15^ Furthermore, within the same tumour, drug‐resistant clones evolve unique Kla signatures that enable their survival under therapeutic pressure, which are distinguishable from those of drug‐sensitive clones.^16^ These heterogeneous Kla patterns expand the complexity of Kla‐driven tumour drug resistance and highlight the need for a more refined understanding of Kla beyond cancer‐type‐specific mechanisms.

Kla, as an emerging research focus in this field, provides groundbreaking perspectives for deciphering the complex mechanisms of tumour drug resistance. In‐depth exploration of its specific roles in drug resistance processes, including its spatial and clonal heterogeneous features within the same tumour, will not only enhance our systematic understanding of tumour resistance networks but also establish a crucial theoretical foundation for elucidating its molecular mechanisms and developing targeted therapeutic strategies. This review aims to comprehensively summarise the molecular mechanisms of Kla modification, systematically elaborate its roles in tumour drug resistance (including DNA damage repair, epigenetic regulation and TME remodelling), explore the spatial and clonal heterogeneity of Kla in the same tumour and its functional implications for drug resistance, analyse the characteristics and mechanisms of Kla in specific cancer types under different resistance contexts and discuss Kla‐targeted therapeutic strategies along with their current challenges and future directions. By providing valuable references for both fundamental research and clinical treatment of tumour drug resistance, this work seeks to advance the development of cancer therapeutics. Notably, this review further constructs a clear spatioclonally evolutionary model of Kla modification across hypoxic tumour core, oxygen‐rich tumour periphery, CTCs and drug‐resistant clones, systematically clarifying the dynamic evolutionary trajectory of Kla profiles during tumour progression and therapeutic stress. Meanwhile, we put forward forward‐looking research concepts including quantitative evaluation systems of intratumoural Kla heterogeneity and the correlation between Kla heterogeneity threshold and clinical treatment response, providing novel theoretical frameworks for subsequent basic research and precise clinical intervention.

## 2 MOLECULAR MECHANISMS OF Kla IN TUMOUR CELLS

Kla is a reversible lysine PTM that uses lactyl‐coenzyme A (CoA) as the donor. Lactyl‐CoA is produced from glycolysis derived lactate through ACSS2. The modification is dynamically controlled by writers including p300/CBP, KAT2A, AARS1/AARS2, erasers including SIRT1–3, HDAC1–3 and readers including TRIM33, DNMT3A. This system regulates histone and non‐histone Kla. It mediates transcriptional reprogramming, DNA damage repair, immunosuppressive TME formation and anti‐apoptotic pathway activation. It drives tumour progression and drug resistance.

### 2.1 Definition and fundamental process of Kla

Kla refers to a covalent PTM wherein a lactyl group is attached to the ε‐amino group (─NH_2_) of lysine residues via esterification. This biochemical process utilises lactyl‐CoA as the primary donor substrate, whereby lactate molecules accumulated via glycolytic pathways are enzymatically transferred to target proteins, resulting in the formation of lactylated lysine. Chemically classified as an acylation modification, Kla shares mechanistic parallels with acetylation but utilises lactate rather than acetyl groups as substrates. This distinction leads to unique alterations in protein structure and charge state, thereby impacting protein conformation, functionality and biological activity.^5^

The modification exhibits high specificity for nucleophilic sites (particularly ε‐amino groups) on lysine residues^17^ and occurs across diverse protein classes including histones (e.g., H3K18la) and non‐histone proteins (e.g., p53, cGAS). These modifications exert regulatory effects in diverse physiological and pathological processes.^11^, ^17^ Kla exists in multiple isomeric forms, predominantly l‐lactylation (KL‐la) in eukaryotic cells and d‐lactylation (KD‐la) in prokaryotes,^18^, ^19^ with subtle chemical environmental differences at modification sites that influence functional outcomes. Notably, a key regulatory feature is the competition between Kla and acetylation for binding at specific histone residues, which creates complex interactions in gene expression (e.g., elevated Kla may suppress acetylation‐mediated transcriptional activation).^20^ This competitive dynamics underscores the intricate crosstalk between metabolic reprogramming and epigenetic regulation in cellular processes.

**FIGURE 1:**
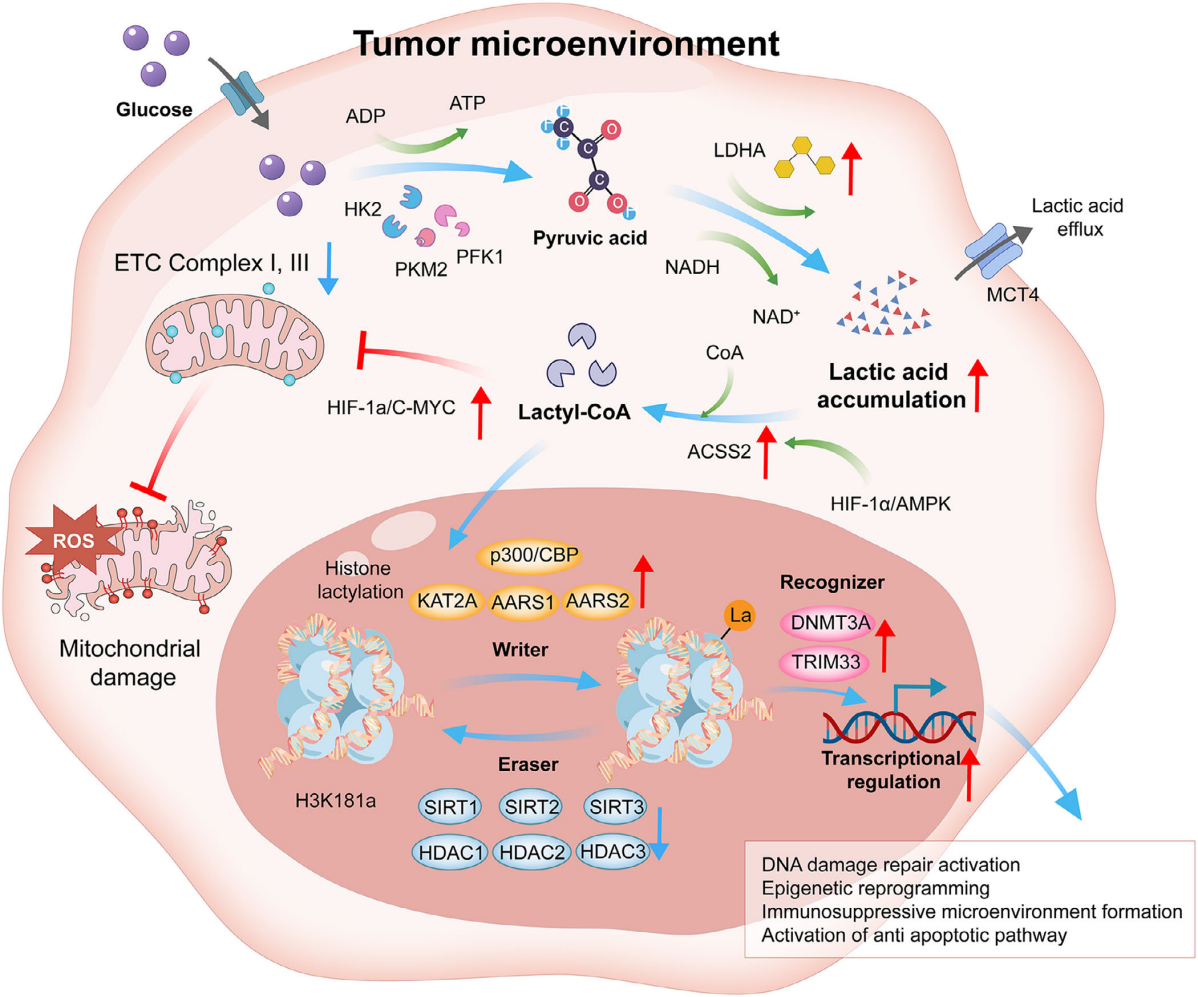
The molecular architecture and core regulatory framework of protein lysine lactylation (Kla).

Kla constitutes a dynamically reversible PTM centrally regulated through the coordinated interplay of lactyltransferases and delactylases, with this enzymatic balance being critically modulated by cellular metabolic status (e.g., lactate concentration), enzyme activity and microenvironmental factors (notably hypoxia). Key lactyltransferases, including p300/CBP and TIP60, catalyse the transfer of lactyl groups from lactyl‐CoA donors to lysine residues on target proteins, frequently sharing substrate specificity with acetyltransferases. Conversely, delactylases mediate the removal of lactyl modifications to achieve reversible regulation. Current evidence indicates histone deacetylases HDAC1–3 and sirtuins (SIRT1–3) execute hydrolytic delactylation, with their enzymatic activity being regulated by lactate concentrations and cellular redox status. For instance, hypoxia‐induced lactate accumulation potently inhibits delactylase activity, and the dynamic regulatory network of Kla is summarised in Figure 1. This schematic illustrates the core interplay between lactyltransferases (writers) and delactylases (erasers), providing a visual framework that contextualises the subsequent analysis of Kla mechanisms.^4^, ^21^, ^22^

### 2.2 Relationship between tumour cell metabolism and Kla

The Warburg effect refers to the preferential reliance of tumour cells on glycolysis over oxidative phosphorylation (OXPHOS) for ATP production, even under oxygen‐replete conditions, which results in excessive lactate accumulation.^23^ This metabolic reprogramming is orchestrated by multiple key regulators, and Table 1 systematically combs the primary functions and regulatory characteristics of these core regulators, which lays a solid molecular foundation for clarifying the regulatory link between Warburg effect‐mediated lactate accumulation and Kla modification.^24^ (1) Lactate dehydrogenase A (LDHA), frequently up‐regulated by oncogenes like MYC, catalyses the reduction of pyruvate to lactate in the final glycolytic step, directly driving lactate accumulation. (2) Coordinated action of glycolytic enzymes, including activated hexokinase 2 (HK2), phosphofructokinase‐1 (PFK1) and pyruvate kinase M2 (PKM2), accelerates the breakdown of glucose to pyruvate.^25^ Notably, isoform switching of PKM2 limits pyruvate entry into mitochondria, further promoting lactate generation. (3) Monocarboxylate transporters (MCTs, e.g., MCT4) export intracellular lactate, maintaining an acidic microenvironment conducive to tumour invasion.^23^ (5) Hypoxia‐inducible factor 1α (HIF‐1α), stabilised under normoxic conditions by aberrantly activated PI3K/AKT/mTOR or Ras pathways, transcriptionally up‐regulates glycolytic enzymes to enhance lactate production.^26^ (6) Oncogene activation (e.g., c‐MYC binding to the LDHA promoter), p53 mutation/loss‐of‐function (reducing OXPHOS capacity) and AMP‐activated protein kinase (AMPK) inactivation (common in tumours) collectively exacerbate the Warburg effect and sustained lactate accumulation.^27^

**TABLE 1:** Key regulators of the Warburg effect and functional characteristics.

Regulator	Primary function	Regulatory characteristics
Lactate dehydrogenase A (LDHA)	Catalyses pyruvate‐to‐lactate conversion, directly driving lactate accumulation	Up‐regulated by oncogenes (e.g., MYC); high expression promotes pyruvate flux to lactate; oxidises NADH to NAD^+^ to sustain glycolysis
Hexokinase 2 (HK2)	Phosphorylates glucose to initiate glycolysis, accelerating pyruvate production	Cooperates with PFK1 and PKM2 to enhance glycolytic rate
Phosphofructokinase‐1 (PFK1)	Rate‐limiting enzyme catalysing fructose‐6‐phosphate phosphorylation	Synergises with HK2 and PKM2 to amplify glycolytic flux
Pyruvate kinase M2 (PKM2)	Converts phosphoenolpyruvate to pyruvate; tetrameric instability limits mitochondrial entry.	Enhanced activity drives glycolysis; isoform switching promotes lactate generation over oxidative phosphorylation.
Monocarboxylate transporters (MCTs, e.g., MCT4)	Exports intracellular lactate, maintaining acidic pH	Facilitates tumour invasion and acidifies the TME
Hypoxia‐inducible factor 1α (HIF‐1α)	Up‐regulates glycolytic enzyme genes; modulates ACSS2 activity	Stabilised by PI3K/AKT/mTOR or Ras pathways under normoxia; regulates lactoyl‐CoA synthesis enzymes in response to metabolic cues
c‐MYC	Binds LDHA promoter to enhance transcription; up‐regulates LDHA expression	Directly drives Warburg effect and exacerbates lactate production
p53 (mutated/inactivated)	Reduces oxidative phosphorylation capacity, reinforcing glycolytic dependency	Indirectly promotes glycolysis and lactate overproduction
AMPK (inactivated)	Sustains lactate accumulation; regulates ACSS2 expression	Frequently inactivated in tumours, relieving lactate production inhibition; participates in lactoyl‐CoA synthesis regulation
GLUT transporters	Mediates glucose uptake for glycolysis	Ensures substrate supply for glycolytic initiation
Lactoyl‐CoA synthetase (e.g., ACSS2)	Catalyses ATP‐dependent lactoyl‐CoA formation from lactate and CoA	Activity enhanced by elevated lactate under HIF‐1α/AMPK regulation; provides high‐energy intermediates for lysine lactylation (Kla)
Transferases (e.g., p300/CBP, TIP60)	Covalently attaches lactyl groups to lysine residues (e.g., H3K18la)	Lactate concentration dependent; competes with acetyl‐CoA pathway; establishes epigenetic feedback regulating Warburg effect

Tumour spatial heterogeneity directly shapes the metabolic gradient of lactate within the TME, thereby driving divergent Kla profiles between the tumour core and periphery. The hypoxic tumour core is the primary site of glycolytic lactate production: HIF‐1α is highly stabilised here, driving the overexpression of LDHA, HK2 and MCT4, which leads to extreme lactate accumulation (up to 20 mM in some solid tumours) and a highly acidic microenvironment (pH 6.0–6.5).^22^, ^28^ This creates a substrate‐rich niche for Kla, resulting in global hyper‐Kla of both histone and non‐histone proteins in core tumour cells. In contrast, the tumour periphery is exposed to relatively sufficient oxygen supply (normoxia or mild hypoxia), with partial tumour cells retaining OXPHOS capacity and lower glycolytic activity.^20^ Lactate concentration in the peripheral TME is significantly lower (2–5 mM), and Kla levels are relatively moderate, with a biased modification pattern towards non‐histone proteins involved in cell migration and invasion (e.g., CXCR4, β‐catenin).^1^ This spatial divergence of Kla is a direct consequence of the metabolic heterogeneity of the TME and forms the basis for the functional specialisation of tumour cells in different spatial regions.

Excessive lactate generation provides tumour cells with rapid ATP production, maintains redox balance (through NAD^+^ regeneration) and establishes an acidic TME conducive to invasion and immune evasion.^23^ Crucially, this process is subject to feedback regulation via epigenetic modifications such as histone Kla, forming a self‐perpetuating cycle. During the lactate generation phase, glucose enters cells through GLUT transporters and undergoes sequential enzymatic conversion to pyruvate via HK, PFK1 and PKM2,^29^ subsequently, pyruvate is reduced to lactate by LDHA while oxidising NADH to NAD^+^ (sustaining glycolytic flux). Enhanced activity of this step under the Warburg effect drives lactate pool expansion. In the lactate activation phase, lactate combines with CoA catalysed by lactoyl‐CoA synthetases (e.g., ACSS2) to form lactyl‐CoA; ACSS2 expression is regulated by metabolic signals (e.g., HIF‐1α or AMPK), with its activity increasing upon lactate accumulation.^30^ This high‐energy intermediate lactyl‐CoA then serves as the lactyl group donor for subsequent modifications. During the Kla modification phase, lactyl‐CoA is recognised by transferases (e.g., p300/CBP or TIP60), which catalyse esterification to covalently attach the lactyl group to the ε‐amino group of target lysine residues (e.g., histone H3K18la)^8^ – a process dependent on cellular lactate concentration and subject to substrate competition with the acetyl‐CoA pathway, and the functional characteristics of each key regulator are detailed in Table 1. This table serves as a molecular roadmap, highlighting how specific regulators (e.g., LDHA, HIF‐1α) serve as the mechanistic basis for lactate accumulation and subsequent Kla.

### 2.3 Feedback regulation of tumour cell metabolism by Kla

Kla orchestrates a metabolic feedback loop in tumour cells, where enhanced glycolysis drives lactate accumulation. This lactate then acts as a substrate for Kla modifications, which reinforce the dominance of glycolysis while suppressing mitochondrial function – thereby contributing to tumour progression (e.g., proliferation, invasion and immune evasion). This self‐reinforcing positive feedback loop operates through epigenetic mechanisms that directly or indirectly regulated the expression and activity of glycolytic enzymes.

This Kla‐driven metabolic feedback loop exhibits spatial specificity within the tumour: in the hypoxic core, histone Kla (e.g., H3K18la, H3K9la) is globally elevated, driving the overexpression of almost all glycolytic enzymes (LDHA, HK2, GLUT1, PKM2) and forming a robust positive feedback loop that locks tumour cells into a glycolysis‐dependent phenotype.^22^ This hyper‐Kla feedback not only sustains high lactate production in the core but also further exacerbates hypoxia by reducing oxygen consumption via mitochondrial dysfunction, creating a self‐amplifying ‘hypoxia–glycolysis–Kla’ cycle. In the tumour periphery, the feedback loop is more moderate and selective: Kla primarily modifies non‐histone proteins (e.g., PKM2, HIF‐1α) rather than global histones, and only up‐regulates glycolytic enzymes associated with cell motility (e.g., MCT1, GLUT3) and lactate efflux, supporting the invasive and metastatic potential of peripheral tumour cells without inducing global glycolytic reprogramming.^1^ This spatial specificity of the Kla feedback loop further reinforces the functional and metabolic heterogeneity of tumour cells in different spatial regions.

Histone Kla (e.g., H3K18la) enhances chromatin accessibility at promoter regions, facilitating transcription factor (TF) binding to up‐regulate key glycolytic genes (e.g., LDHA, HK2, GLUT1).^31^ Elevated H3K18la levels in tumour cells directly increase LDHA expression, amplifying pyruvate‐to‐lactate conversion.^32^, ^33^ Concurrently, non‐histone Kla directly modulates glycolytic enzymes, such as PKM2 Kla inhibiting its tetrameric active conformation, triggering pyruvate accumulation and increased glycolytic flux.^34^ Furthermore, Kla stabilises and activates TFs (e.g., HIF‐1α, c‐MYC) to amplify glycolytic signalling.^31^ Stabilised HIF‐1α subsequently induces GLUT1 and PFK1 expression, accelerating glucose uptake and metabolism.^35^

This self‐perpetuating cycle entrenches tumour cells in glycolytic reliance under high‐lactate conditions, maintaining the Warburg effect through a vicious loop. Excessive lactate production enhances Kla, which in turn induces up‐regulation of glycolytic enzymes, ultimately leading to further lactate accumulation. This process simultaneously acidifies the TME and confers proliferative advantages. Crucially, Kla suppresses mitochondrial OXPHOS to reinforce glycolytic commitment through dual mechanisms: (1) direct targeting of mitochondrial proteins (e.g., electron transport chain complexes I and III) alters their activity and stability.^36^ For example, Kla of complex I subunits reduces electron transfer efficiency, diminishing ATP synthesis while increasing reactive oxygen species (ROS) production and causing mitochondrial damage. (2) Transcriptional reprogramming down‐regulates mitochondrial biogenesis genes (e.g., PGC‐1α) while up‐regulating glycolytic genes (e.g., LDHA), suppressing mitochondrial renewal and function.^30^ This metabolic rewiring diverts pyruvate towards lactate production rather than the tricarboxylic acid (TCA) cycle. (3) Kla‐enhanced HIF‐1α activity inhibits respiratory chain gene expression and promotes glycolysis‐mitochondria uncoupling,^37^ thereby reducing mitochondrial membrane potential and activating mitophagy – ultimately compromising mitochondrial integrity. Consequently, Kla‐driven mitochondrial dysfunction forces tumour cells into obligatory glycolysis, facilitating hypoxia adaptation and driving invasion–metastasis cascades.

**FIGURE 2:**
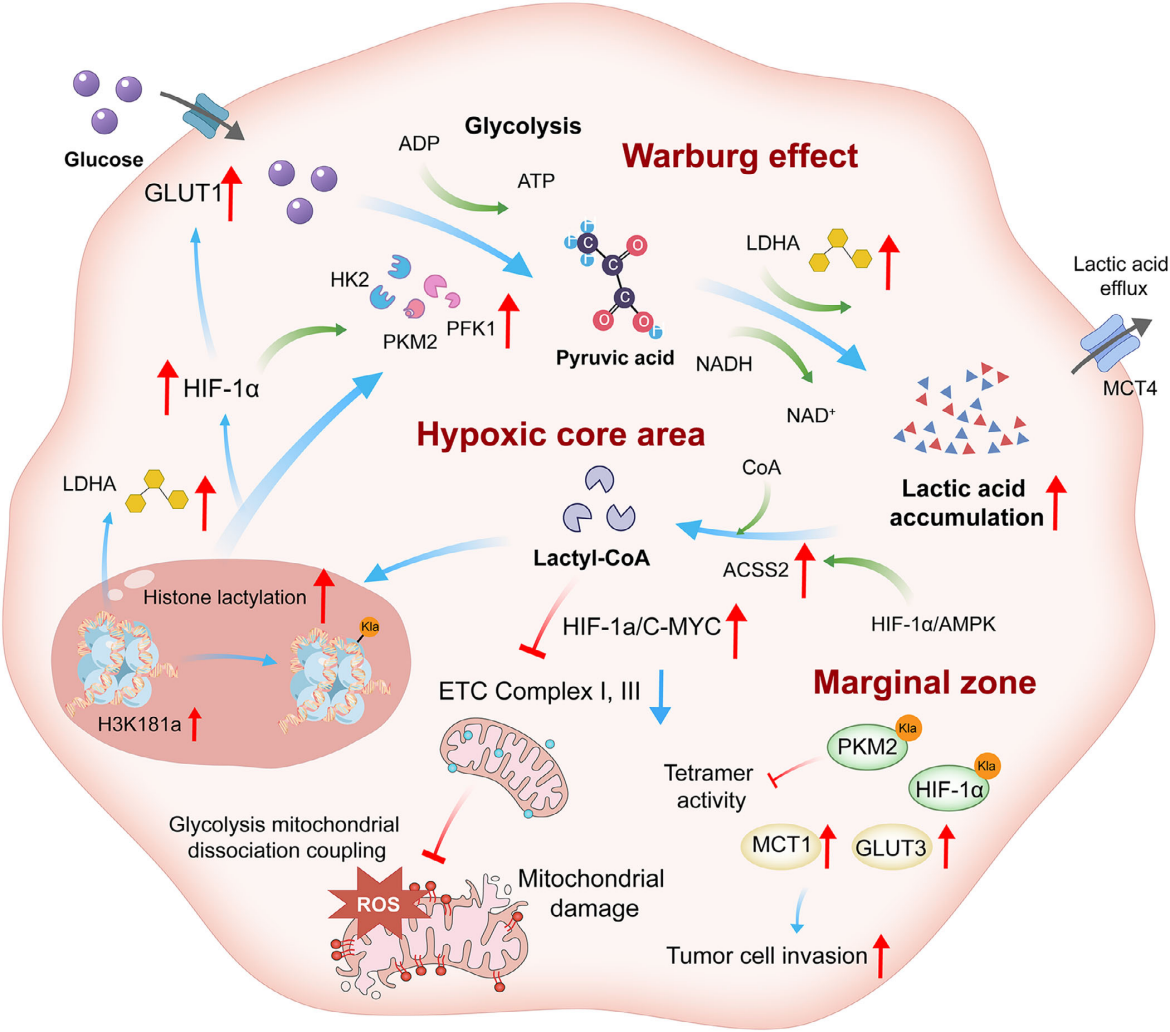
Metabolic–epigenetic feedback loop: lysine lactylation (Kla) sustains the Warburg effect and glycolytic reprogramming.

Collectively, Kla functions as a central feedback regulator in tumour cell metabolism by augmenting the expression of glycolytic enzymes while suppressing mitochondrial function, thereby sustaining metabolic reprogramming (i.e., Warburg effect) and conferring survival advantages. This pivotal role establishes Kla as a promising therapeutic target, as exemplified by the development of lactyltransferase inhibitors, and Figure 2 provides a comprehensive visualisation of this Kla‐driven metabolic feedback loop. As depicted, the figure delineates the dual regulatory arms of Kla: its role in enhancing glycolysis and suppressing mitochondrial OXPHOS, thereby mechanistically explaining the formation of the Warburg effect positive feedback loop.

Glycolysis in tumour cells generates abundant lactate, which is converted to lactyl‐CoA by ACSS2 to drive histone Kla. Kla up‐regulates glycolytic genes, stabilises HIF‑1α and suppresses mitochondrial OXPHOS, forming a self‑reinforcing cycle that promotes Warburg effect, tumour invasion and therapeutic resistance.

### 2.4 The classical writer–eraser–reader regulatory framework of Kla

As a typical reversible PTM, Kla follows a well‐established canonical writer–eraser–reader regulatory paradigm that has been widely recognised in epigenetic research, which is strictly controlled by writer (lactyltransferase), eraser (delactylase) and reader (Kla‐binding protein) proteins, which jointly control the dynamic modification level of Kla on histone and non‐histone substrates, and further regulates the downstream biological processes such as gene transcription, protein function and metabolic reprogramming in tumour cells.^38^ This regulatory framework is the core molecular basis for Kla to exert biological effects, and its abnormal activation is closely related to the occurrence of tumour drug resistance.^39^

The writer–eraser–reader regulatory framework of Kla, which has been established as a mature and conserved paradigm in epigenetic modification research, is not only dysregulated in tumour cells compared with normal cells but also exhibits distinct activation patterns across different spatial regions of the tumour and different clonal populations. In the hypoxic tumour core, lactyltransferases (e.g., p300/CBP, KAT2A) are highly activated, while delactylases (e.g., SIRT2, HDAC1–3) are significantly inhibited due to high lactate levels and hypoxia, leading to a net increase in Kla levels.^39^ In the tumour periphery, lactyltransferase activity is moderate and delactylases retain partial function, resulting in a dynamic balance of Kla that is responsive to microenvironmental changes. Furthermore, drug‐resistant tumour clones exhibit a constitutively activated writer–eraser balance (e.g., persistent p300/CBP activation and SIRT2 down‐regulation) that maintains high Kla levels even under therapeutic pressure, while drug‐sensitive clones show a labile balance that is easily disrupted by anti‐tumour therapies. CTCs, on the other hand, display a unique writer–eraser–reader profile adapted to the circulatory microenvironment, with up‐regulation of specific lactyltransferases (e.g., AARS1/AARS2) and readers (e.g., TRIM33) that support their survival in the bloodstream.^1^, ^22^

#### 2.4.1 Kla writers: lactyltransferases with substrate specificity

Lactyltransferases (writers) are the core enzymes that catalyse the formation of Kla modification, which transfer the lactyl group from the key donor lactyl‐CoA to the ε‐amino group of lysine residues on target proteins. To date, multiple functional lactyltransferases have been identified and validated in tumour cells, with p300/CBP, KAT2A and AARS1/AARS2 being the most classical and well‐characterised writers, and their catalytic activities are tightly regulated by tumour microenvironmental lactate concentration and metabolic signals. p300/CBP is the first identified histone lactyltransferase, which not only catalyses the Kla of histone lysine residues (e.g., H3K18la, H3K9la) in tumour cells, but also mediates the Kla modification of non‐histone proteins (e.g., BCL‐XL, STAT3, HIF‐1α).^40^ Its lactyltransferase activity is dependent on the binding of lactyl‐CoA, and the high lactate microenvironment in tumours can significantly enhance its catalytic efficiency, thereby promoting the accumulation of Kla modification on oncogenic and drug resistance‐related proteins. p300/CBP is the dominant lactyltransferase in the hypoxic tumour core, mediating global histone Kla, while KAT2A is preferentially activated in the tumour periphery, catalysing Kla of histones associated with cell invasion genes. AARS1/AARS2 is highly expressed in CTCs, acting as a lactate sensor and mediating Kla of DNA damage repair proteins (e.g., p53, cGAS) to support CTC survival in the circulation. KAT2A acts as a lactyltransferase by coupling with ACSS2 (lactyl‐CoA synthetase), and the ACSS2–KAT2A complex can specifically catalyse histone Kla in the nucleus of tumour cells and is involved in the regulation of lactate metabolism‐related gene transcription, forming a metabolic–epigenetic feedback loop.^30^ AARS1 and AARS2 are newly discovered non‐histone lactyltransferases with lactate‐sensing properties, which can sense the intracellular lactate concentration and catalyse the Kla of key proteins such as p53 and cGAS, and participate in the regulation of DNA damage repair and innate immune response in tumour cells, thus becoming an important link between Kla and tumour drug resistance.^17^ In addition, TIP60 has also been confirmed to have lactyltransferase activity in tumour cells, which shares partial substrate specificity with p300/CBP and is involved in the Kla modification of histone H4 and other substrates, jointly regulating the open state of chromatin and the transcription of drug resistance‐related genes.^2^

#### 2.4.2 Kla erasers: delactylases mediating modification reversal

Delactylases (erasers) are enzymes that catalyse the removal of lactyl groups from modified lysine residues, which reverse Kla modification and restore the original structure and function of target proteins, thus forming a dynamic balance with lactyltransferases. The currently identified delactylases in tumour cells are mainly members of the SIRT family and class I HDACs, and their enzymatic activity is regulated by tumour metabolic status (e.g., lactate concentration, redox balance) and hypoxic microenvironment. SIRT1, SIRT2 and SIRT3 are NAD^+^‐dependent deacetylases with delactylase activity, among which SIRT2 is the most studied tumour suppressor delactylase in Kla regulation.^36^ It can specifically remove the lactyl group of histone H3K18la and H3K9la in tumour cells, restore the expression of tumour suppressor genes (e.g., p53) and inhibit the expression of glycolytic enzymes (e.g., HK2, PFKFB3), thus breaking the Warburg effect and Kla feedback loop. SIRT2 activity is almost completely inhibited in the hypoxic tumour core and drug‐resistant clones, leading to the accumulation of Kla modifications, while it retains partial activity in the tumour periphery and drug‐sensitive clones, limiting excessive Kla. SIRT3 is the dominant delactylase in CTCs, mediating the delactylation of mitochondrial proteins to maintain partial mitochondrial function and support CTC survival under nutrient limitation. SIRT1 and SIRT3 mainly mediate the delactylation of mitochondrial proteins and non‐histone proteins (e.g., cyclin E2, LDHA), and participate in the regulation of mitochondrial metabolism and cell cycle progression in tumour cells. HDAC1, HDAC2 and HDAC3 (class I HDACs) are the first identified histone delactylases, which can hydrolytically remove the lactyl group on histone lysine residues in a lactate concentration‐dependent manner. Hypoxia‐induced lactate accumulation in tumours can significantly inhibit the delactylase activity of HDAC1–3, leading to the accumulation of histone Kla modification and the up‐regulation of downstream oncogene transcription.^9^ The delactylase activity of the above erasers is the key to maintaining the dynamic balance of Kla in tumour cells. The down‐regulation or functional inactivation of erasers (e.g., SIRT2 down‐regulation in advanced tumours) is a common molecular event in tumour drug resistance, which leads to the abnormal accumulation of Kla modification and the persistent activation of drug resistance‐related signalling pathways. Drug‐resistant clones exhibit a permanent down‐regulation of delactylases (SIRT2, HDAC2) at the genetic or epigenetic level, in contrast to drug‐sensitive clones where delactylase down‐regulation is only transient and reversible under metabolic stress.^24^, ^41^

#### 2.4.3 Kla readers: potential binding proteins mediating functional output

Kla readers (readers) are proteins that specifically recognise and bind to lactylated lysine residues on target proteins, and transduce the Kla modification signal into downstream biological effects, which are the key links for Kla to exert epigenetic and post‐translational regulatory functions. At present, the research on Kla readers is still in the exploratory stage, but several potential reader proteins have been identified in tumour cells through lactylome screening and pull‐down experiments, and their binding specificity and functional characteristics have been initially validated. TRIM33 has been reported as a Kla‐specific reader protein, which can specifically bind to lactylated histone residues (e.g., H3K18la) in tumour cells, and recruit transcriptional repressor complexes to regulate the transcription of target genes, thus participating in the regulation of tumour cell proliferation and apoptosis.^42^ DNA methyltransferases (DNMTs; including DNMT3A) are speculated to serve as potential Kla readers by recognising histone Kla modification, though direct experimental evidence remains lacking. DNMT3A can specifically bind to H3K18la and H3K27la on the promoter of tumour suppressor genes (e.g., p53, PUMA) in tumour cells and mediate DNA hypermethylation of the promoter region, leading to the silencing of tumour suppressor gene expression and the occurrence of drug resistance. Kla readers exhibit distinct binding specificities in different tumour spatial regions and clonal populations: TRIM33 is the dominant reader in the hypoxic tumour core, binding H3K18la to silence tumour suppressor genes, while DNMT3A is preferentially recruited in drug‐resistant clones, mediating epigenetic silencing of pro‐apoptotic genes. In CTCs, bromodomain‐containing proteins (BRD4) act as key Kla readers, recognising lactylated histones associated with stemness genes (e.g., OCT4, SOX2) to maintain CTC stemness and metastatic potential.^43^ TF co‐activators/co‐repressors (e.g., p300, HDAC3) also have potential Kla reading activity. They can recognise the Kla modification on their own or other proteins and regulate their own enzymatic activity or protein–protein interaction, thus forming a self‐regulatory loop of Kla modification. In addition, some bromodomain‐containing proteins (BRD) and plant homeodomain finger proteins have been shown to have potential Kla‐binding activity in in vitro experiments, which are considered as candidate Kla readers in tumour cells.^44^ The in‐depth identification and functional verification of Kla readers will be the key to revealing the downstream molecular mechanism of Kla in tumour drug resistance, especially the specific reader proteins that mediate Kla signals in CTCs and drug‐resistant clones.

#### 2.4.4 Synergistic regulation of the writer–eraser–reader framework in tumour drug resistance

In tumour cells, the writer, eraser and reader of Kla form a synergistic regulatory network, which takes lactate metabolism as the core and mediates the occurrence of tumour drug resistance through multiple dimensions. High lactate concentration in the TME not only enhances the lactyltransferase activity of writers (e.g., p300/CBP) to promote Kla accumulation, but also inhibits the delactylase activity of erasers (e.g., HDAC1–3, SIRT2) to reduce modification removal, leading to the abnormal enrichment of Kla on histone and non‐histone substrates.^9^, ^45^ The accumulated Kla modification is then specifically recognised by readers (e.g., TRIM33, DNMT3A), which further regulates the transcription of drug resistance‐related genes (e.g., BCL2, PD‐L1, ABCB1) and the function of key proteins (e.g., NBS1, MRE11, AKT),^46^ ultimately leading to the activation of DNA damage repair, epigenetic reprogramming, immunosuppressive microenvironment formation and anti‐apoptosis pathways in tumour cells.^42^, ^47^ The disruption of this regulatory network (e.g., overexpression of writers, inactivation of erasers, abnormal binding of readers) is a common molecular basis for the development of drug resistance in various tumours (e.g., breast cancer, lung cancer, pancreatic cancer), and targeting the key components of this framework has become a promising therapeutic strategy for overcoming tumour drug resistance.

This synergistic regulatory network of the well‐recognised canonical writer–eraser–reader framework for Kla exhibits profound heterogeneity in the same tumour, which is a key driver of intratumoural drug resistance diversity: (1) in the hypoxic tumour core, the network is constitutively activated, maintaining global hyper‐Kla and driving intrinsic drug resistance of core tumour cells to chemotherapy and radiation; (2) in the tumour periphery, the network is dynamically regulated, and Kla levels can be further elevated under therapeutic pressure, leading to the acquisition of drug resistance in peripheral tumour cells; (3) CTCs possess a unique writer–eraser–reader network adapted to the circulatory microenvironment, which mediates the drug resistance of CTCs to systemic therapy and supports their metastatic potential; (4) drug‐resistant clones within the tumour evolve a stably dysregulated writer–eraser–reader network (e.g., p300/CBP overexpression, SIRT2 deletion, DNMT3A hyperactivation) that forms a Kla signature distinct from drug‐sensitive clones, enabling their selective survival under therapeutic pressure. These heterogeneous regulatory patterns of the Kla framework mean that a single Kla‐targeted therapy cannot effectively eliminate all tumour cell populations, and personalised and combinatorial therapeutic strategies based on intratumoural Kla heterogeneity are required.^48^

## 3 Kla‐DRIVEN MECHANISMS OF TUMOUR DRUG RESISTANCE

Kla promotes tumour drug resistance through four interrelated biological processes, including DNA damage repair activation, epigenetic regulation and gene expression reprogramming, TME remodelling and immune escape, as well as imbalance between cellular survival and apoptosis. This section systematically elaborates the core molecular mechanisms of Kla in mediating tumour drug resistance from the above four dimensions, with a clear and progressive logical relationship between subsections, so as to fully demonstrate the key role of Kla in the formation of drug‐resistant phenotypes. DNA damage repair, epigenetic regulation,^49^ TME remodelling^50^ and cell survival–apoptosis balance^51^: each subsection will focus on answering the core scientific question of how Kla modifies key molecules in the corresponding process to drive the formation of drug resistance phenotype. Notably, all these mechanisms exhibit spatial and clonal heterogeneity within the tumour, and the Kla‐driven drug resistance effects in the tumour core, periphery, CTCs and drug‐resistant clones are distinct and complementary,^52^ jointly contributing to the overall drug resistance of the tumour.

### 3.1 Activation of DNA damage repair pathways

This section addresses how Kla modifies key proteins in DNA damage repair pathways to enhance the repair capacity of tumour cells for therapy‐induced DNA damage, thereby reducing the cytotoxic effect of anti‐tumour drugs and leading to drug resistance. Elevated lactate levels in the TME trigger activation of key DNA damage response pathways (e.g., ATM/ATR signalling) via Kla modifications Acting as a signalling molecule, lactate promotes the recruitment of repair proteins (e.g., RAD51, Ku70/80) to DNA double‐strand break (DSB) sites, accelerating the initiation of repair processes. This enables cancer cells to counteract therapy‐induced DNA damage, thereby enhancing their survival and driving treatment resistance. For instance, Kla of the DNA repair protein NBS1 at specific lysine residues (e.g., K388) stabilises the MRN complex, expediting its recruitment to DSBs and facilitating ATM phosphorylation. This cascade activates downstream effectors (e.g., CHK2, p53), boosting homologous recombination (HR) efficiency and DSB repair capacity 11. Consistently, recent studies have identified Kla of MRE11 (another core component of the MRN complex) at K673, which further strengthens MRN complex assembly and HR‐mediated DSB repair, synergising with NBS1 Kla to enhance therapeutic resistance.^53^ Such enhanced repair reduces genomic instability while increasing tolerance to DNA‐damaging therapies. Notably, Kla also modulates the activity of poly(ADP‐ribose) polymerase 1 (PARP1), a key mediator of single‐strand break (SSB) repair and PARP inhibitor sensitivity; Kla of PARP1 at its automodification domain enhances its catalytic activity, promoting SSB repair and reducing the efficacy of PARP inhibitors in HR‐proficient tumours.^54^ Additionally, Kla improves repair accuracy by positively regulating HR (e.g., promoting RAD51 foci formation), conferring resistance to HR‐dependent therapies like PARP inhibitors.^53^ Beyond DSB repair, Kla exerts an additional regulatory role in SSB repair: in ALDH1A3‐overexpressing glioblastoma cells, lactate‐driven Kla of XRCC1 at lysine 247 (K247) neutralises its surface charge, thereby enhancing its interaction with importin α and subsequent nuclear translocation; this process reinforces SSB repair capacity and confers resistance to radiotherapy and temozolomide.^55^

The Kla‐mediated activation of DNA damage repair pathways exhibits significant spatial and clonal heterogeneity^1^: (1) the hypoxic tumour core shows the most robust activation of this mechanism, with hyper‐Kla of NBS1, MRE11, PARP1 and XRCC1, leading to ultra‐efficient DNA damage repair and intrinsic resistance to DNA‐damaging therapies (e.g., cisplatin, radiotherapy)^11^; (2) the tumour periphery has moderate Kla of DNA repair proteins, and its repair capacity is only significantly enhanced under therapeutic pressure, leading to acquired resistance; (3) drug‐resistant clones possess constitutive Kla of key DNA repair proteins (e.g., NBS1 K388la, MRE11 K673la) at the clonal level, which is a stable genetic/epigenetic feature and not affected by microenvironmental changes, making these clones highly resistant to DNA‐damaging therapies^53^; (4) CTCs exhibit selective Kla of DNA repair proteins associated with oxidative stress damage (e.g., XRCC1, PARP1), as they encounter high levels of ROS in the circulatory system, and this Kla‐mediated repair capacity enables CTCs to resist ROS‐induced DNA damage and survive in the bloodstream. In contrast, drug‐sensitive clones show only transient Kla of DNA repair proteins under metabolic stress, and their repair capacity is easily disrupted by anti‐tumour therapies, leading to cell death.

**FIGURE 3:**
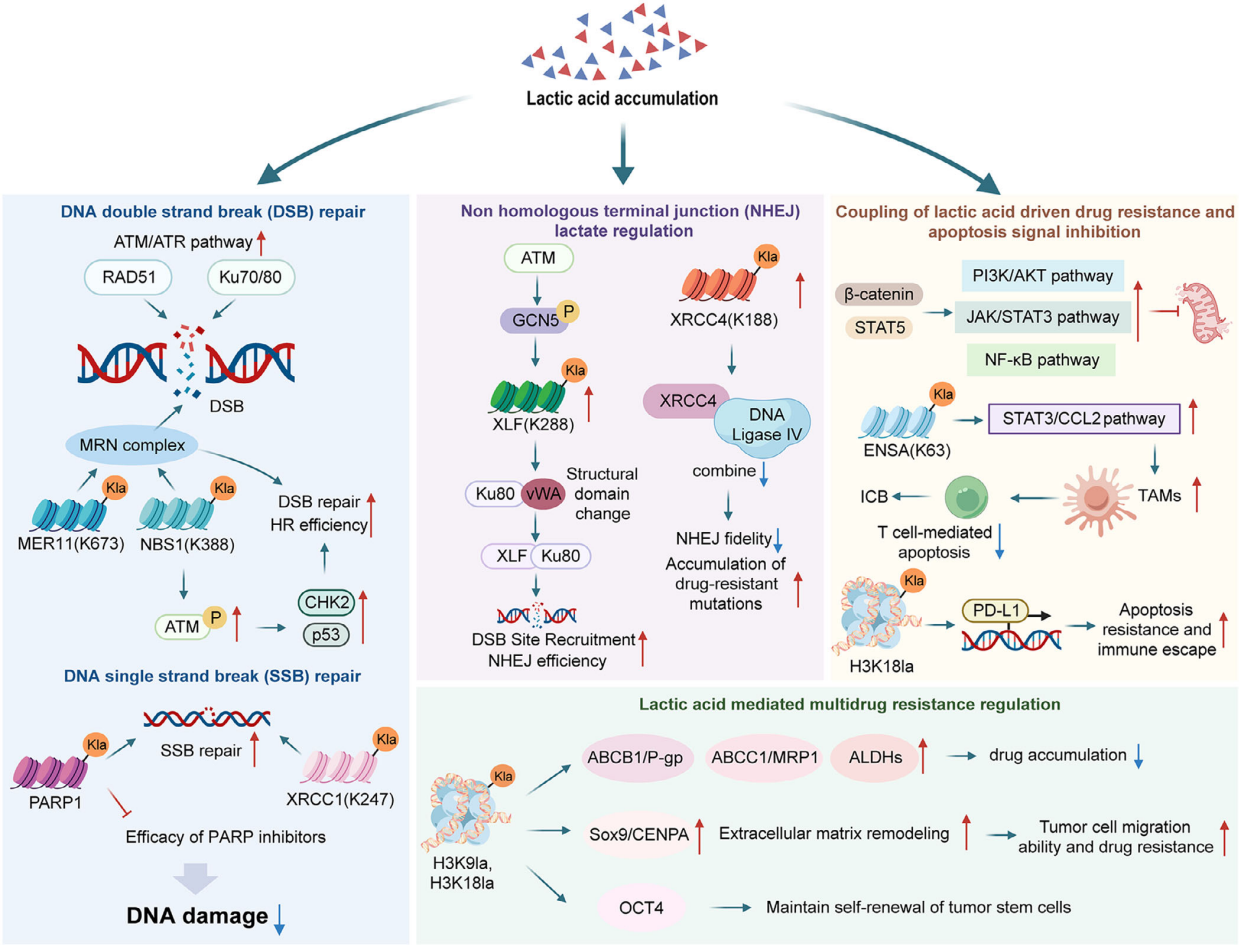
Lysine lactylation (Kla)‐mediated DNA damage repair and genomic stability.

In contrast, Kla exerts complex, context‐dependent regulation on non‐homologous end joining (NHEJ) – ranging from suppression to bidirectional modulation – thereby increasing error rates. A recent study (2025) revealed that lactate induces Kla of XLF (a core NHEJ scaffold protein) at K288 within its Ku‐binding motif; this modification is driven by ATM‐mediated phosphorylation of GCN5, which enhances GCN5–XLF interaction and XLF Kla.^56^ Lactylated XLF forms a more stable complex with Ku80 via conformational changes in the Ku80 vWA domain, promoting XLF recruitment to DSB sites and enhancing NHEJ efficiency, which confers resistance to chemotherapeutics such as 5‐fluorouracil and oxaliplatin.^56^ While this rapid but error‐prone repair aids cancer cell survival under stress, it may accelerate tumour evolution. Furthermore, Kla of XRCC4 (another NHEJ core protein) at K118 impairs its binding to DNA ligase IV, reducing NHEJ fidelity and promoting accumulation of drug‐resistant mutations.^53^ Critically, Kla‐driven drug resistance is tightly coupled to the suppression of apoptotic signalling, forming a dual protective mechanism for cancer cells. Kla activates pro‐survival pathways (e.g., PI3K/AKT, JAK/STAT3, NF‐κB) by modifying key regulators such as β‐catenin and STAT5, which directly inhibit the intrinsic mitochondrial apoptotic pathway.^53^ Additionally, Kla reshapes the tumour immune microenvironment to indirectly suppress apoptosis: lactate‐induced K63 Kla of endosulfine α (ENSA–K63la) triggers STAT3/CCL2 signalling, promoting recruitment of tumour‐associated macrophages (TAMs) and formation of an immunosuppressive microenvironment that impairs cytotoxic T cell‐mediated apoptosis, leading to immune checkpoint blockade (ICB) resistance.^46^ At the epigenetic level, histone Kla (e.g., H3K18la) up‐regulates the transcription of anti‐apoptotic genes and immune checkpoint molecules (e.g., PD‐L1), further reinforcing apoptosis resistance and immune escape.^53^ Collectively, Kla disrupts the HR/NHEJ balance to exacerbate therapeutic resistance. Its regulatory scope extends beyond DNA repair and apoptosis to multiple key resistance mechanisms: (1) epigenetic reprogramming: histone Kla (e.g., H3K9la, H3K18la) up‐regulates the expression of drug efflux pumps (e.g., ABCB1/P‐gp, ABCC1/multi‐drug resistance protein 1 [MRP1]) and detoxifying enzymes (e.g., ALDHs), reducing intracellular drug accumulation; (2) epithelial–mesenchymal transition (EMT): Kla promotes Sox9/CENPA overexpression and extracellular matrix remodelling via hypoxia–Kla crosstalk, enhancing tumour cell motility and drug resistance; (3) cancer stem cells (CSCs) maintenance: Kla modifies OCT4 and other stemness regulators, sustaining CSCs self‐renewal and multi‐drug resistance (MDR).^53^ These findings highlight Kla as a central integrator of metabolic reprogramming and epigenetic regulation in tumour drug resistance. The disruption of the HR/NHEJ balance by Kla is more pronounced in drug‐resistant clones and the tumour core,^1^ which not only leads to therapeutic resistance but also accelerates the accumulation of genetic mutations in these cell populations, further driving tumour evolution and the development of MDR.^11^, ^53^ The intricate mechanisms by which Kla modifies DNA damage repair proteins to promote drug resistance are schematically illustrated in Figure 3.

Kla promotes the repair of DNA DSBs and SSBs by modifying key repair proteins including NBS1, MRE11, PARP1, XRCC1, XLF and XRCC4. It enhances HR efficiency and reduces the fidelity of non‐homologous end joining, thereby conferring resistance to DNA‐damaging chemotherapies and radiotherapy. Meanwhile, Kla activates pro‐survival signalling and inhibits apoptosis, forming a dual mechanism that drives therapeutic resistance.

### 3.2 Epigenetic regulation and gene expression reprogramming

This section addresses how Kla regulates chromatin structure and the activity of key TFs through histone and non‐histone Kla, respectively, to drive epigenetic reprogramming and abnormal gene expression in tumour cells, and thus mediate the formation of drug resistance. This section is divided into two parts: the regulatory effect of histone Kla on chromatin structure and gene transcription,^57^ and the regulatory effect of non‐histone Kla on TF activity, to systematically elaborate the epigenetic regulatory mechanism of Kla in tumour drug resistance.^58^ This epigenetic reprogramming driven by Kla exhibits distinct spatial and clonal patterns: the tumour core undergoes global histone Kla and extensive epigenetic reprogramming related to glycolysis and anti‐apoptosis,^40^ the tumour periphery undergoes selective epigenetic reprogramming related to invasion and metastasis, CTCs undergo epigenetic reprogramming related to stemness and survival^59^ and drug‐resistant clones undergo stable epigenetic reprogramming related to MDR, all of which are mediated by divergent Kla modification profiles.

#### 3.2.1 Impact of histone Kla on chromatin structure and gene transcription

Kla of lysine residues (Kla), primarily occurring on histones H3 (e.g., H3K18, H3K9) and H4, is driven by elevated lactate levels in the TME.^30^ This modification neutralises the positive charge of lysine, thereby weakening histone–DNA interactions, promoting nucleosome disassembly and enhancing chromatin accessibility. Concurrently, Kla competes with acetylation for the same lysine sites and inhibits HDAC activity, collectively maintaining an open chromatin state conducive to TF binding. Notably, recent studies have revealed that Kla exhibits distinct enrichment patterns at active enhancers in addition to promoters, and frequently colocalises with the classic enhancer marker H3K27ac to form ‘super Kla regions (SLRs)’, dense Kla clusters that drive hypertranscription of tissue‐specific oncogenes.^60^ In T‐cell acute lymphoblastic leukaemia (T‐ALL), these SLRs are selectively enriched in the regulatory regions of anti‐apoptotic genes (e.g., BCL2) and oncogenic TFs (e.g., NOTCH1, TAL1), reinforcing the anti‐apoptotic phenotype and therapeutic resistance.^60^ Consequently, Kla activates the transcription of drug resistance‐associated genes such as MDR1/P‐gp and BCL2, enhancing drug efflux capacity and anti‐apoptotic activity in cancer cells. Histone Kla and the formation of SLRs exhibit striking spatial and clonal heterogeneity: (1) in the hypoxic tumour core, SLRs are widely distributed in the regulatory regions of glycolytic genes (LDHA, HK2), anti‐apoptotic genes (BCL2, MCL1) and DNA damage repair genes (NBS1, MRE11), driving the hypertranscription of these genes and forming intrinsic drug resistance^11^, ^40^; (2) in the tumour periphery, SLRs are only enriched in the regulatory regions of invasion and metastasis‐related genes (VIM, SNAI1, CXCR4), promoting tumour cell motility and distant metastasis without inducing global drug resistance; (3) drug‐resistant clones possess unique SLRs that are enriched in the regulatory regions of MDR genes (ABCB1, ABCC1, PD‐L1), which is a stable clonal signature and can be inherited during cell division, making these clones resistant to multiple therapeutic modalities^51^; (4) CTCs form SLRs in the regulatory regions of stemness genes (OCT4, SOX2, NANOG) and anti‐oxidative stress genes (SOD2, GPx4), maintaining CTC stemness and enabling their survival in the circulatory system.^49^, ^59^ In contrast, drug‐sensitive clones lack stable SLRs, and their histone lactylation is only transiently induced under metabolic stress, with no persistent epigenetic reprogramming.^1^ Mechanistically, this process is tightly regulated by a lactyl‐CoA synthetase axis: nuclear‐localised GTPSCS catalyses lactate into lactyl‐CoA, and its interaction with p300 (a key histone acetyltransferase/lactyltransferase) is mediated by acetylation of GTPSCS at K73, specifically promoting H3K18la modification and transcriptional activation of downstream targets including GDF15, a gene closely linked to immune escape and anti‐apoptosis in glioma.^61^ Furthermore, in specific chromatin regions, Kla recruits repressive proteins including DNMTs, leading to the silencing of tumour suppressor genes like p53.^8^ Recent evidence (2023–2025) indicates that Kla‐induced recruitment of DNMT3A not only silences p53 but also targets the pro‐apoptotic gene PUMA, whose promoter hypermethylation reduces its expression and impairs apoptotic signalling in colorectal cancer (CRC).^62^, ^63^ Additionally, H3K27la has been identified as a novel repressive modification that binds the promoter of the pro‐apoptotic gene BIM, recruiting HDAC3‐containing repressive complexes to suppress BIM transcription, thereby conferring chemoresistance in lung and breast cancer.^64^ Beyond direct regulation of apoptotic genes, histone Kla intersects with other epigenetic modifications to fine‐tune apoptosis and drug resistance: H3K18la synergises with H3K27ac at the promoter of the anti‐apoptotic gene Survivin, amplifying its transcription and inhibiting caspase activation,^19^ while in glioblastoma, H3K18la‐mediated up‐regulation of GDF15 reshapes the tumour immune microenvironment, suppressing cytotoxic T cell‐induced apoptosis and enhancing resistance to ICB.^61^ Moreover, non‐histone Kla of epigenetic regulators expands this regulatory network – Kla of the histone methyltransferase EZH2 at K124 stabilises its binding to chromatin, promoting H3K27me3‐mediated silencing of CASP3 (a key executioner caspase) and contributing to sorafenib resistance in hepatocellular carcinoma.^19^ The dynamic balance of histone Kla is also modulated by de‐lactylases: members of the KDAC family (e.g., SIRT1, SIRT2) exhibit de‐lactylase activity, and down‐regulation of SIRT2 in advanced tumours leads to accumulation of H3K9la and H3K18la, which sustain the silencing of pro‐apoptotic genes and enhance therapeutic resistance.^19^ This ‘writer–eraser’ regulatory axis highlights the reversibility of Kla‐mediated apoptotic suppression, offering potential therapeutic targets. Together, these mechanisms enhance cancer cell tolerance to chemotherapy and targeted therapy via chromatin opening‐mediated up‐regulation of drug resistance genes and silencing of tumour suppressor genes. The recruitment of DNMT3A by histone Kla is a key feature of drug‐resistant clones, where this process leads to the permanent epigenetic silencing of pro‐apoptotic and tumour suppressor genes,^1^, ^65^ in contrast to the transient silencing observed in drug‐sensitive clones under metabolic stress.

#### 3.2.2 Regulation of TF activity by non‐histone Kla

Kla directly or indirectly targets lysine residues on key TFs, including HIF‐1α, STAT3 and MYC, catalysing their modification. This Kla inhibits ubiquitination‐mediated degradation (e.g., HIF‐1α K394la), thereby prolonging TF half‐life and enabling sustained activation of downstream genes.^17^ Notably, recent studies (2023–2025) have identified additional Kla sites on HIF‐1α, such as K532la, which enhances its interaction with the lactyltransferase p300, forming a positive feedback loop that amplifies HIF‐1α transcriptional activity and up‐regulates the anti‐apoptotic gene BNIP3 – this further suppresses mitochondrial apoptotic pathway activation under hypoxic conditions and strengthens resistance to DNA‐damaging chemotherapies (e.g., cisplatin).^32^, ^66^, ^67^ Concurrently, Kla alters protein conformation (e.g., STAT3 K49la), modulating DNA‐binding affinity to either enhance or diminish transcriptional efficiency. Mechanistically, this conformational change is often coordinated with other PTMs: STAT3 K49la synergises with Y705 phosphorylation to promote dimerisation and nuclear retention, while also inhibiting the recruitment of phosphatases that terminate STAT3 signalling, thereby sustaining the transcription of anti‐apoptotic genes (e.g., BCL2, MCL1) and immune checkpoint molecules (e.g., PD‐L1).^46^, ^68^ Non‐histone Kla of TFs exhibits selective modification patterns in different tumour cell populations: (1) HIF‐1α is hyper‐lactylated at multiple sites (K394la, K532la) in the tumour core, driving the sustained activation of glycolytic and anti‐apoptotic genes^69^; (2) STAT3 is preferentially lactylated at K49la in the tumour periphery and TAM‐infiltrated regions, promoting the transcription of immune checkpoint molecules (PD‐L1) and chemokines (CCL2), leading to immune evasion^70^; (3) MYC is constitutively lactylated at K323la in drug‐resistant clones, driving the overexpression of drug efflux pumps and anti‐apoptotic genes, forming MDR^71^; (4) CTCs exhibit Kla of SOX2 and OCT4 at specific sites, sustaining their stemness and metastatic potential. In drug‐sensitive clones, the Kla of these TFs is transient and can be reversed by therapeutic interventions, leading to the inactivation of downstream resistance genes.^72^ For example, HIF‐1α Kla activates genes such as VEGF and GLUT1, promoting glycolysis and angiogenesis to support tumour growth,^11^ while STAT3 Kla up‐regulates anti‐apoptotic genes including BCL2 and MCL1, inhibiting apoptosis and contributing to resistance against immunotherapy.^73^ Beyond HIF‐1α, STAT3 and MYC, Kla has been shown to target additional TFs critical for apoptosis and drug resistance: in pancreatic ductal adenocarcinoma (PDAC), lactate‐induced K63 Kla of ENSA is a regulatory protein that modulates TF activity triggers STAT3/CCL2 signalling, recruiting TAMs to form an immunosuppressive microenvironment that impairs cytotoxic T cell‐mediated apoptosis and drives ICB resistance^46^; in CRC, β‐catenin K49la prevents its proteasomal degradation, and the stabilised β‐catenin forms a nuclear complex with FOXO3a to suppress the transcription of pro‐apoptotic genes (e.g., BIM, PUMA), conferring resistance to PI3K/AKT inhibitors.^74^ For MYC, a key oncogenic TF, recent research (2024) reveals that lactate‐derived Kla at K323 enhances its binding to the co‐activator MAX, specifically up‐regulating the transcription of drug efflux pumps (e.g., ABCB1/P‐gp) and the anti‐apoptotic gene Survivin – this dual effect reduces intracellular drug accumulation and inhibits caspase‐3 activation, leading to MDR against paclitaxel and 5‐fluorouracil in breast and lung cancer.^19^ Additionally, MYC Kla intersects with metabolic reprogramming by up‐regulating LDHA expression, further increasing lactate production and creating a ‘lactate–MYC Kla’ positive feedback loop that sustains anti‐apoptotic signalling and metabolic addiction.^32^ The regulatory network of TF Kla is further refined by specific ‘writer’ and ‘eraser’ enzymes: p300, previously identified as a histone lactyltransferase, also mediates non‐histone Kla of STAT3 and HIF‐1α, while KDAC family members (e.g., SIRT5) function as de‐lactylases that reverse MYC K323la and β‐catenin K49la, down‐regulation of SIRT5 in advanced tumours leads to accumulation of these lactylated TFs and exacerbation of apoptotic resistance.^19^, ^75^ Moreover, the lactyl‐CoA synthetase GTPSCS generates nuclear lactyl‐CoA, a critical substrate for TF Kla, and its overexpression in glioma promotes STAT3 Kla and subsequent up‐regulation of GDF15, a key mediator of immune escape and apoptotic suppression.^61^ Collectively, by facilitating persistent TF activation, this mechanism drives anti‐apoptotic signalling and metabolic reprogramming, allowing cancer cells to survive therapeutic pressure. The expanding repertoire of lactylated TFs, their context‐dependent modification sites and crosstalk with metabolic and epigenetic pathways highlight Kla as a central hub integrating transcriptional regulation, apoptosis suppression and multi‐modal therapeutic resistance, from chemotherapy and targeted therapy to immunotherapy.

Kla remodels chromatin accessibility and TF activity via histone and non‐histone modifications. Histone Kla (H3K9la, H3K18la, H3K27la) forms super Kla regions to drive oncogene and drug‐resistance gene transcription, while silencing tumour suppressor genes. Kla of HIF‐1α, STAT3, MYC and β‐catenin stabilises these TFs to sustain anti‐apoptotic, immune‐evasive and MDR programs. These epigenetic events exhibit spatial and clonal heterogeneity and directly promote therapeutic resistance.

### 3.3 Remodelling of the TME and immune escape

This section addresses how Kla modifies key molecules in immune cells and tumour cells within the TME to reshape the TME into an immunosuppressive state, thereby enabling tumour cells to evade immune surveillance and attack and leading to resistance to immunotherapy and other anti‐tumour therapies. This section is divided into two parts: the regulation of TAM polarisation by Kla and the up‐regulation of immune checkpoint protein expression by Kla,^46^, ^76^ to elaborate the regulatory mechanism of Kla in TME remodelling and immune escape.^77^, ^78^ Notably, all Kla‐mediated TME remodelling and immune escape mechanisms exhibit pronounced spatial and clonal heterogeneity within the same tumour: the hypoxic tumour core forms a constitutively immunosuppressive microenvironment driven by hyper‐Kla, the tumour periphery displays inducible immunosuppression under therapeutic pressure, CTCs evolve a unique Kla‐dependent immune escape profile adapted to the circulatory microenvironment, and drug‐resistant clones possess a stable Kla signature that mediates intrinsic immunosuppression,^1^ all of which jointly contribute to the overall immunotherapy resistance of the tumour.

#### 3.3.1 Impact of Kla modification on TAM polarisation

**FIGURE 4:**
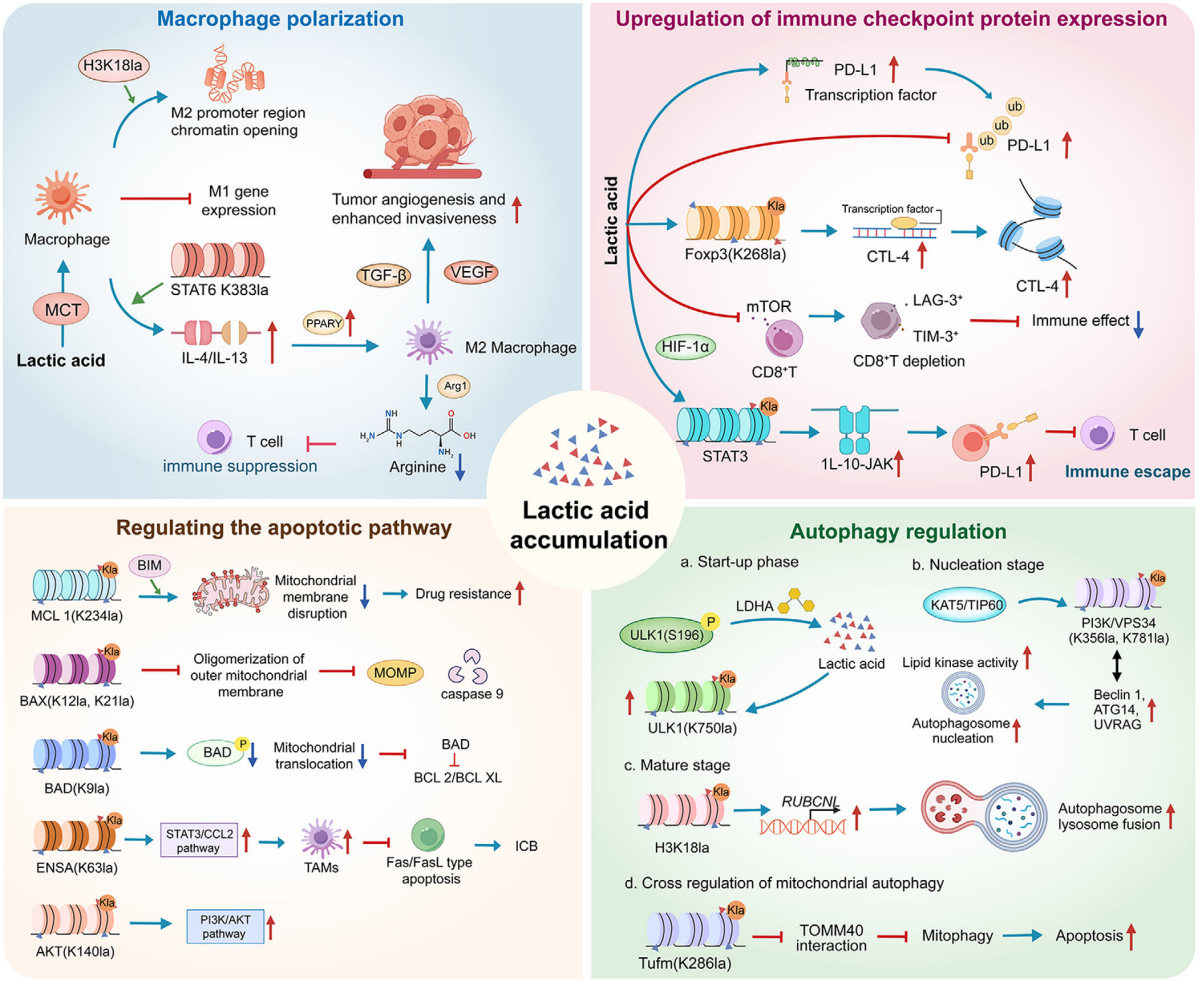
Imbalance in cell survival–apoptosis axis and tumour microenvironment remodelling.

Elevated lactate, produced by tumour cells via glycolysis, is primarily taken up by macrophages through MCTs.^79^ This triggers Kla modifications, such as H3K18la on histone H3, which promote an open chromatin state at the promoters of M2‐type genes while concurrently repressing M1‐type gene expression.^80^ Additionally, Kla modification at the K383 site of STAT6 (STAT6 K383la) enhances signal transduction through the IL‐4/IL‐13 pathway, activating the TF PPARγ and ultimately driving macrophage polarisation towards the M2 phenotype.^81^ M2‐polarised TAMs secrete cytokines, including TGF‐β and VEGF, which promote tumour angiogenesis and enhance the invasive capacity of cancer cells.^82^ Regarding immunosuppression, M2 TAMs release arginase‐1 (Arg1), which depletes arginine within the TME, thereby suppressing T cell proliferation and function.^83^ Consequently, these processes collectively increase the proportion of M2 TAMs within the TME. This shift impairs the response to chemotherapy and immunotherapy, compromising the efficacy of therapeutic agents such as PD‐1 inhibitors and CTLA‐4 antibodies, and the intricate regulatory network governing M2 macrophage polarisation is schematised in Figure 4.

Spatially, the hypoxic tumour core is the primary site of Kla‐driven M2 macrophage polarisation: the extreme lactate accumulation here induces global H3K18la in TAMs, driving constitutive M2 polarisation and persistent secretion of Arg1 and TGF‐β, forming a ‘permanent’ immunosuppressive niche that is inherently resistant to immunotherapy. In contrast, the tumour periphery has moderate lactate levels, and macrophage Kla (STAT6 K383la) is only significantly induced under therapeutic pressure (e.g., chemotherapy or immunotherapy), leading to acquired M2 polarisation and inducible immunosuppression that can be partially reversed by reducing lactate levels.^84^, ^85^ Clonally, drug‐resistant clones secrete high levels of lactate through a hyper‐activated Warburg effect, which induces specific Kla of TAMs in their microenvironment (e.g., PU.1 K23la) that is not observed in drug‐sensitive clones, enhancing the adhesion of TAMs to drug‐resistant clones and forming a ‘protective barrier’ against immune cell attack. CTCs, on the other hand, induce Kla of monocytes in the circulatory system (e.g., H3K9la) through lactate secretion, driving their differentiation into M2‐like macrophages that accompany CTCs to distant metastatic sites, providing an immunosuppressive microenvironment for CTC colonisation and metastasis.^86^

#### 3.3.2 Mechanisms underlying Kla‐mediated up‐regulation of immune checkpoint protein expression

Kla‐mediated up‐regulation of immune checkpoint proteins involves multiple targets, including PD‐L1 on tumour cells, CTLA‐4 on T cells and LAG‐3 on immune cells, with regulatory mechanisms encompassing tumour cell PD‐L1 elevation, T cell CTLA‐4/LAG‐3 activation, Kla modification and myeloid cell PD‐L1 expression.^12^ Specifically, HIF‐1α enhances PD‐L1 stability and binds its promoter, increasing PD‐L1 transcription 2–3‐fold,^87^ while lactate can directly modify PD‐L1, inhibiting its ubiquitination‐mediated degradation to promote immune escape.^88^ Furthermore, Kla of Foxp3 (K268la) enhances its DNA binding affinity, promoting CTLA‐4 transcription,^89^ and lactate inhibits mTOR signalling in CD8^+^ T cells, inducing an exhausted phenotype (LAG‐3^+^TIM‐3^+^) that reduces immunotherapy response rates.^90^ Finally, STAT3 Kla activates the IL‐10–JAK pathway, up‐regulating PD‐L1 on monocytes, suppressing T cell activation and ultimately establishing an immunosuppressive TME, and Figure 4 comprehensively depicts the dual‐axis regulatory network of Kla, which simultaneously drives macrophage polarisation and immune checkpoint up‐regulation to establish an immunosuppressive microenvironment.

This Kla‐mediated immune checkpoint up‐regulation exhibits distinct spatial and clonal characteristics: (1) in the hypoxic tumour core, tumour cells display hyper‐Kla of PD‐L1 (K282la) and H3K18la‐driven PD‐L1 transcriptional up‐regulation, resulting in extremely high PD‐L1 expression levels that form a strong immune checkpoint barrier; meanwhile, CD8^+^ T cells in the core undergo severe Kla‐induced exhaustion (LAG‐3^+^TIM‐3^+^TIGIT^+^) due to high lactate exposure, losing cytotoxic function irreversibly.^58^ (2) In the tumour periphery, tumour cell PD‐L1 Kla is transient and only up‐regulated under therapeutic pressure, and CD8^+^ T cell exhaustion is mild and partially reversible, making the periphery a potential target for immunotherapy combination strategies.^84^, ^91^ (3) Drug‐resistant clones possess a unique Kla signature of immune checkpoint molecules: PD‐L1 K282la is a stable clonal marker that is not affected by microenvironmental changes, and Foxp3 K268la in regulatory T cells (Tregs) within the clone microenvironment is constitutively activated, leading to persistent CTLA‐4 up‐regulation and intrinsic immunotherapy resistance.^1^, ^85^ (4) CTCs exhibit specific Kla of PD‐L1 (K162la) that is distinct from primary tumour cells, which enhances PD‐L1 binding to PD‐1 on circulating immune cells and enables CTCs to evade immune surveillance in the bloodstream; additionally, CTCs induce Kla of LAG‐3 on NK cells, inhibiting NK cell‐mediated cytotoxicity and further promoting CTC survival and metastasis.^92^

### 3.4 Imbalance between cellular survival and apoptotic pathways

This section addresses how Kla disrupts the balance between cell survival and apoptosis by modifying key molecules in the apoptotic pathway and autophagy process, thereby enabling tumour cells to resist therapy‐induced apoptosis and maintain survival and leading to drug resistance. This section will elaborate on the regulatory effect of Kla on the intrinsic mitochondrial apoptotic pathway, extrinsic death receptor pathway and autophagy process, and clarify the mechanism of Kla in mediating the imbalance of cell survival and apoptosis. Kla‐mediated disruption of the survival–apoptosis balance is a core mechanism of tumour drug resistance, and this process exhibits significant spatial and clonal heterogeneity within the same tumour: the tumour core has hyper‐Kla‐driven constitutive apoptosis resistance,^5^, ^91^ the tumour periphery has inducible apoptosis resistance under stress, CTCs evolve a Kla‐dependent survival mechanism adapted to nutrient limitation and oxidative stress in the circulation and drug‐resistant clones possess a stable Kla signature that mediates intrinsic apoptosis resistance to multiple therapies, which is a key clonal marker distinguishing them from drug‐sensitive clones.

Under conditions of elevated lactate in the TME, Kla primarily targets specific lysine residues in BCL‐2 family proteins. This modification blocks mitochondrial outer membrane permeabilisation (MOMP), thereby preventing cytochrome c release and inhibiting caspase cascade activation.^93^ Recent studies (2023–2025) have identified specific Kla sites in key BCL‐2 family members: for anti‐apoptotic protein MCL‐1, Kla at K234 stabilises its interaction with pro‐apoptotic BIM, neutralising BIM‐mediated mitochondrial membrane disruption and enhancing resistance to BH3 mimetics (e.g., venetoclax) in acute myeloid leukaemia,^94^ for pro‐apoptotic protein BAX, Kla at K12 and K21 inhibits its oligomerisation on the mitochondrial outer membrane, directly blocking MOMP initiation and caspase‐9 activation in CRC.^95^ Additionally, Kla of BAD at K9 impairs its dephosphorylation and translocation to mitochondria, preventing BAD from antagonising BCL‐2/BCL‐XL and sustaining the anti‐apoptotic state.^96^ Concurrently, Kla activates the PI3K/AKT pathway, up‐regulating BCL‐2 expression and establishing a positive feedback loop.^97^ Mechanistically, Kla of AKT at K140 enhances its membrane localisation and phosphorylation, while H3K18la‐mediated up‐regulation of USP39 stabilises PGK1 to further amplify PI3K/AKT/HIF‐1α signalling, forming a ‘Kla–PI3K/AKT–BCL‐2’ axis that reinforces apoptotic resistance in endometrial and pancreatic cancer.^95^ This cascade of events confers resistance to chemotherapy‐induced apoptosis in tumour cells, consequently enhancing their survival. Beyond the intrinsic mitochondrial pathway, Kla also disrupts the extrinsic death receptor pathway: in PDAC, lactate‐induced K63 Kla of ENSA (ENSA–K63la) activates STAT3/CCL2 signalling, recruiting TAMs to form an immunosuppressive microenvironment that impairs cytotoxic T cell‐mediated Fas/FasL‐dependent apoptosis, leading to ICB resistance.^46^ Direct Kla of caspase‐3 at K14 has also been identified as a key inhibitory mechanism, this modification blocks caspase‐3 activation by preventing its cleavage into active subunits, thereby suppressing apoptosis induced by chemotherapy (e.g., doxorubicin) in ALL.^98^

Spatially, the hypoxic tumour core exhibits the most robust Kla‐mediated apoptosis resistance: core tumour cells display global hyper‐Kla of anti‐apoptotic proteins (BCL‐2 K7la, MCL‐1 K234la) and severe Kla inhibition of pro‐apoptotic proteins (BAX K12la, caspase‐3 K14la), forming a ‘double barrier’ against both intrinsic and extrinsic apoptotic pathways; additionally, the PI3K/AKT pathway in core cells is constitutively activated by AKT K140la, further reinforcing the anti‐apoptotic state.^58^ In contrast, the tumour periphery has moderate Kla levels, with only transient modification of BCL‐2 family proteins under therapeutic pressure, and the apoptotic pathway can be partially reactivated by inhibiting Kla, making the periphery more sensitive to pro‐apoptotic therapies.^84^, ^91^ Clonally, drug‐resistant clones possess constitutive Kla of key anti‐apoptotic molecules as a stable genetic/epigenetic feature: MCL‐1 K234la and caspase‐3 K14la are specific Kla markers of drug‐resistant clones, which are not present in drug‐sensitive clones even under metabolic stress; this stable Kla signature enables drug‐resistant clones to resist various pro‐apoptotic therapies (chemotherapy, targeted therapy, immunotherapy).^98^ CTCs, adapting to the circulatory microenvironment, exhibit selective Kla of anti‐oxidative stress and anti‐apoptotic proteins (e.g., SOD2 K68la, BCL‐XL K122la), which not only resists ROS‐induced apoptosis in the bloodstream but also maintains cell survival under glucose and nutrient limitation, a Kla profile that is completely distinct from primary tumour cells.^92^

Additionally, autophagy supports tumour cell survival by clearing damaged proteins and organelles to maintain energy homeostasis and redox balance. During chemotherapy or nutrient deprivation, autophagy provides an alternative energy source, enabling tumour cells to evade therapy‐induced cell death and ultimately contributing to resistance against multiple therapeutic modalities. Furthermore, Kla modification can directly or indirectly regulate key autophagy proteins, inducing autophagy initiation, enhancing autophagosome formation efficiency, promoting autophagosome–lysosome fusion and ultimately augmenting autophagic flux.^99^ Notably, Kla targets core autophagy regulators with precise mechanisms: (1) initiation: ULK1 phosphorylates LDHA at S196 to promote lactate production, which in turn mediates ULK1 Kla at K750, enhancing its kinase activity and autophagy initiation under nutrient deprivation^93^, ^99^; (2) nucleation: acyltransferase KAT5/TIP60 mediates Kla of PI3K/ VPS34 at K356 and K781, strengthening its interaction with Beclin‐1, ATG14 and UVRAG to increase lipid kinase activity and autophagosome nucleation^93^; (3) maturation: histone H3K18la directly up‐regulates the transcription of RUBCNL, a key autophagy enhancer that promotes autophagosome–lysosome fusion, conferring resistance to bevacizumab in CRC^99^; (4) mitophagy crosstalk: Kla of Tufm at K286 inhibits its interaction with TOMM40, suppressing mitophagy and promoting mitochondrial dysfunction‐induced apoptosis evasion in tumour cells.^100^ Critically, Kla coordinates the crosstalk between apoptosis and autophagy to maximise tumour cell survival: by inhibiting apoptotic signalling while enhancing cytoprotective autophagy, Kla creates a ‘dual survival barrier’.^94^ For example, in glioblastoma, Kla‐induced autophagy clears damaged mitochondria to reduce ROS accumulation, thereby preventing ROS‐mediated caspase activation; conversely, autophagy can degrade pro‐apoptotic proteins (e.g., BAX) to further reinforce apoptotic resistance.^96^, ^99^ This synergistic regulation enables tumour cells to adapt to diverse therapeutic stresses, including chemotherapy, targeted therapy and immunotherapy.

The lactylation‐mediated regulation of autophagy also exhibits distinct spatial and clonal heterogeneity: (1) in the hypoxic tumour core, Kla drives constitutive activation of autophagy: H3K18la‐mediated up‐regulation of RUBCNL and ULK1 K750la‐induced autophagy initiation form a persistent autophagic flux that provides a continuous energy source for core cells under nutrient limitation,^99^ and mitophagy is severely suppressed by Tufm K286la, further enhancing cell survival.^100^ (2) In the tumour periphery, autophagy is only induced by Kla under therapeutic pressure (e.g., chemotherapy‐induced nutrient stress), and the autophagic flux is moderate and reversible, making peripheral cells sensitive to autophagy inhibitors combined with chemotherapy.^84^, ^91^ (3) Drug‐resistant clones possess a unique Kla signature of autophagy proteins: ATG5 K103la, LC3B K49la and p62 K7la are stable clonal markers that drive constitutive high autophagic flux, which is a key mechanism for drug‐resistant clones to clear therapeutic‐induced toxic proteins and maintain survival; this Kla profile of autophagy proteins is not observed in drug‐sensitive clones, which only display transient autophagy protein Kla under acute stress.^58^ (4) CTCs exhibit selective lactylation of autophagy proteins associated with nutrient adaptation (e.g., Beclin‐1 K146la, ATG16L1 K23la), which enhances autophagosome formation under glucose limitation and enables CTCs to utilise alternative energy sources (e.g., fatty acid oxidation [FAO]) in the bloodstream^92^; additionally, CTCs display Kla of mitophagy‐related proteins (e.g., PINK1 K222la) that is distinct from primary tumour cells, which modulates mitophagy to maintain mitochondrial homeostasis under oxidative stress, a critical survival mechanism for CTCs in the circulatory microenvironment.^101^

Kla promotes M2 macrophage polarisation and up‐regulates immune checkpoints (PD‐L1, CTLA‐4) to remodel an immunosuppressive microenvironment. It stabilises anti‐apoptotic proteins (MCL‐1, BCL‐2), inhibits pro‐apoptotic factors (BAX, caspase‐3) and enhances cytoprotective autophagy, thereby disrupting survival–apoptosis balance and driving tumour immune escape and drug resistance.

## 4 Kla IN DIVERSE TUMOUR TYPES AND RESISTANCE CONTEXTS

Section 3 systematically elaborated the universal molecular mechanisms of Kla‐mediated tumour drug resistance from four core aspects: DNA damage repair, epigenetic regulation, TME remodelling and cell survival–apoptosis balance,^102^ and highlighted the spatial and clonal heterogeneity of Kla within the same tumour as a key feature of these mechanisms.^4^ On the basis of these universal mechanisms, this section will conduct a specific case analysis of the Kla‐mediated drug resistance mechanism in four common malignant tumours (breast cancer, CRC, lung cancer, pancreatic cancer), focusing on clarifying the cancer‐specific Kla targets, modified sites and unique regulatory pathways in different tumour types and different resistance contexts, and integrating the tumour‐specific spatial and clonal Kla characteristics into the analysis of each cancer type, to form a logical system of ‘universal mechanism, cancer‐specific characteristics and intratumoural heterogeneity’. Table 2 will systematically compare and summarise the above cancer‐specific Kla‐mediated drug resistance mechanisms,^103^ including the corresponding intratumoural Kla heterogeneity features.^1^

**TABLE 2:** Comparison of lysine lactylation (Kla)‐mediated drug resistance mechanisms in different cancer types.

Cancer type	resistance type	Lysine lactylation (Kla) target(s)	Mediated resistance mechanism	Key signalling pathway(s)
Breast cancer	HER2‐targeted therapy (trastuzumab) resistance	H3K18la	Opens the BCL‐2 promoter; competes with H3K18ac binding and blocks HDAC de‐modification, sustaining high BCL‐2 expression	BCL‐2 pathway: suppresses mitochondrial apoptosis, enabling evasion of ADCC
		AKT1 (K189 site)	Allosterically activates AKT kinase activity, leading to constitutive downstream pathway activation	PI3K/AKT pathway: phosphorylates mTORC1 to inhibit autophagy; phosphorylates FOXO1 to suppress pro‐apoptotic genes
Colorectal cancer	5‐FU chemotherapy resistance	H3K27la	Opens the PD‐L1 promoter (enhancing STAT3 binding); suppresses CXCL9/10 transcription (reducing CD8^+^ T cell infiltration); activates genes for M2 macrophage polarisation and Treg differentiation	Immunosuppressive axis: PD‐L1 up‐regulation, impaired T‐cell infiltration, Treg/M2 polarisation
		Autophagy proteins (ATG5 K103la, LC3B K49la, p62 K7la)	Enhances autophagosome elongation and autophagosome–lysosome fusion; inhibits ubiquitin‐mediated degradation; activates FAO to replenish TCA intermediates	Autophagy flux pathway: ATG5, LC3B, p62; fatty acid oxidation (FAO)
Lung cancer	EGFR‐TKI resistance	H3K9la	Drives epigenetic reprogramming of EMT genes: silences E‐cadherin, up‐regulates Vimentin and activates SNAI1	EMT pathway: E‐cadherin, Vimentin, SNAI1 (enhances invasion, migration and stemness)
		TFAM (K88 site)	Enhances mtDNA transcription (increasing copy number and respiratory chain complexes); activates OXPHOS (boosting ATP generation); up‐regulates antioxidant defences (scavenging ROS, inhibiting ferroptosis)	Mitochondrial metabolism pathway: TFAM, OXPHOS, SOD2/GPx4
Pancreatic cancer	Immunotherapy resistance	H3K122la (in CAFs)	Opens COL1A1/FN1 promoters (promoting collagen/fibronectin deposition and thickening the physical barrier); activates IL‐6/IL‐8 transcription (recruiting MDSCs and Tregs); inhibits LATS1 to activate YAP1 (promoting fibrosis)	TME/stroma pathway: COL1A1/FN1, IL‐6/IL‐8, YAP1
		CXCR4 (K288 site)	Enhances protein stability (doubling half‐life), sustaining CXCL12/CXCR4 axis activation; activates ERK/FAK (increasing motility); activates NF‐κB (up‐regulating PD‐L1)	Metastasis/immune evasion axis: CXCR4, ERK/FAK, PD‐L1

### 4.1 Breast cancer: HER2‐targeted therapy resistance

#### 4.1.1 Association between H3K18 Kla and BCL‐2‐mediated trastuzumab resistance

Elevated lactate levels in the TME induce Kla at lysine 18 of histone H3 (H3K18la). This modification remodels chromatin architecture at the BCL‐2 promoter, enhancing its accessibility to TFs and thereby up‐regulating BCL‐2 expression by 2–3‐fold.^104^ Mechanistically, H3K18la competes with histone acetylation (H3K18ac) for binding at the same residue, which blocks HDAC‐mediated demodification and sustains constitutive BCL‐2 overexpression.^105^ In breast cancer, BCL‐2 overexpression inhibits the mitochondrial apoptosis pathway, enabling tumour cells to evade trastuzumab‐induced antibody‐dependent cellular cytotoxicity (ADCC) and enhancing therapeutic resistance.^106^ Collectively, these findings indicate that H3K18la‐driven BCL‐2 up‐regulation disrupts apoptotic signalling, exacerbating trastuzumab resistance. Therapeutic strategies targeting this axis, such as LDHA inhibitors that reduce H3K18la levels, may down‐regulate BCL‐2 and restore trastuzumab sensitivity, as further consolidated in Table 2, which compares the distinct Kla‐mediated resistance mechanisms across the cancer types discussed in this section.

In breast cancer tumours, H3K18la‐mediated BCL‐2 up‐regulation exhibits distinct spatial heterogeneity: the hypoxic tumour core has extremely high H3K18la levels and BCL‐2 expression, leading to intrinsic trastuzumab resistance; the tumour periphery has moderate H3K18la levels and BCL‐2 expression, and trastuzumab sensitivity can be partially restored by inhibiting Kla.^1^, ^107^ Clonally, trastuzumab‐resistant breast cancer clones possess H3K18la as a stable clonal marker, and the BCL‐2 promoter in these clones has a unique chromatin landscape that is highly susceptible to Kla‐mediated activation, which is not observed in trastuzumab‐sensitive clones; additionally, CTCs from HER2‐positive breast cancer exhibit H3K18la levels that are 2.5‐fold higher than primary tumour cells, leading to high BCL‐2 expression and enabling CTCs to evade trastuzumab‐induced ADCC in the bloodstream, a key mechanism for distant metastasis in trastuzumab‐resistant breast cancer.^108^

#### 4.1.2 Activation of the PI3K/AKT signalling pathway by AKT Kla

In breast cancer, lactate directly induces Kla of AKT1 at lysine 189 (AKT1 K189la). This modification allosterically activates AKT kinase activity, leading to constitutive activation of its downstream signalling cascades.^105^ Specifically, activated AKT promotes tumour cell survival by phosphorylating mTORC1 to inhibit autophagy, while also phosphorylating FOXO1 to suppress pro‐apoptotic gene expression, thereby enhancing cellular anti‐apoptotic capacity.^109^ Persistent PI3K/AKT pathway activation impairs trastuzumab‐mediated endocytic inhibition of HER2 signalling, consequently promoting breast cancer cell proliferation and survival.^110^ Targeting this mechanism, AKT Kla inhibitors can specifically block K189 Kla, restoring trastuzumab's therapeutic efficacy, and the specific molecular targets and signalling pathways involved in this resistance mechanism are detailed in Table 2 for comparative reference.

AKT1 K189la‐mediated PI3K/AKT activation in breast cancer displays pronounced spatial and clonal heterogeneity: (1) spatial heterogeneity: the hypoxic tumour core has constitutive AKT1 K189la and PI3K/AKT activation, leading to persistent HER2 signalling and trastuzumab resistance; the tumour periphery has transient AKT1 K189la under therapeutic pressure, and PI3K/AKT activation is reversible, making the periphery a potential target for combination therapy with AKT inhibitors and trastuzumab.^111^ (2) Clonal heterogeneity: trastuzumab‐resistant clones exhibit AKT1 K189la as a stable clonal marker, with constitutive PI3K/AKT activation that is not affected by trastuzumab treatment; in contrast, trastuzumab‐sensitive clones only display transient AKT1 K189la under metabolic stress, and PI3K/AKT activation can be effectively inhibited by trastuzumab.^1^ (3) CTC heterogeneity: breast cancer CTCs exhibit AKT1 K189la levels that are significantly higher than primary tumour cells, and the PI3K/AKT pathway in CTCs is constitutively activated, which not only enhances CTC survival in the bloodstream but also promotes CTC colonisation at metastatic sites (e.g., lung, bone), a key mechanism for trastuzumab‐resistant distant metastasis.^112^

### 4.2 CRC: 5‐FU chemotherapy resistance

#### 4.2.1 H3K27 Kla‐mediated immune escape mechanisms

Elevated lactate in the TME induces Kla at lysine 27 of histone H3 (H3K27la). In the context of immunosuppressive gene regulation, H3K27la remodels the chromatin architecture of the PD‐L1 promoter region, enhancing its accessibility to STAT3 and thereby up‐regulating PD‐L1 expression. Concurrently, H3K27la occupancy at the promoters of CXCL9 and CXCL10 suppresses their transcription, impairing interferon gamma (IFN‐γ) responsiveness and reducing CD8^+^ T cell infiltration. In myeloid immune cell regulation, H3K27la activates genes associated with M2 macrophage polarisation and promotes Treg differentiation, thereby establishing an immunosuppressive microenvironment.^113^ Clinically, CRC patients exhibit a positive correlation between H3K27la levels and PD‐L1 expression; those with high H3K27la levels show a 4.3‐month shorter progression‐free survival (PFS) following 5‐FU treatment compared with controls. Notably, LDHA inhibitors reduce H3K27la levels, restore T cell infiltration and reverse 5‐FU resistance, highlighting a cancer‐specific mechanism that is systematically compared with other tumour types in Table 2.

In CRC, H3K27la‐mediated immune escape and 5‐FU resistance exhibit distinct intratumoural heterogeneity: (1) spatial heterogeneity: the hypoxic tumour core has extremely high H3K27la levels, leading to constitutive PD‐L1 up‐regulation and severe CD8^+^ T cell infiltration deficiency, forming an immunosuppressive microenvironment that is inherently resistant to 5‐FU combined with immunotherapy; the tumour periphery has moderate H3K27la levels, with partial CD8^+^ T cell infiltration, and LDHA inhibitors can effectively reduce H3K27la levels and restore the sensitivity of peripheral tumour cells to 5‐FU. (2) Clonal heterogeneity: 5‐FU‐resistant CRC clones possess H3K27la as a stable clonal marker, and the PD‐L1 promoter in these clones has a unique Kla‐responsive element that is not present in 5‐FU‐sensitive clones; additionally, Tregs within the microenvironment of 5‐FU‐resistant clones exhibit constitutive Foxp3 K268la, leading to persistent CTLA‐4 up‐regulation and further enhancing immunosuppression.^1^ (3) CTC heterogeneity: CRC CTCs exhibit H3K27la levels that are threefold higher than primary tumour cells, leading to high PD‐L1 expression and enabling CTCs to evade immune surveillance in the bloodstream; additionally, CRC CTCs induce Kla of CXCR4 on immune cells, inhibiting immune cell migration and further promoting CTC survival and distant metastasis.^114^

#### 4.2.2 Role of Kla in promoting autophagy and chemotherapy resistance

In CRC, Kla of key autophagy proteins enhances drug resistance: ATG5 (K103la) increases autophagosome elongation efficiency to clear 5‐FU‐induced toxic proteins.^115^ LC3B (K49la) promotes autophagosome–lysosome fusion, providing energy to sustain cell survival.^116^ Meanwhile, p62 (K7la) inhibits ubiquitin‐mediated degradation, enhances autophagic flux and accelerates regeneration of damaged organelles, and Table 2 systematically categorises the specific Kla targets and functional mechanisms of these autophagy proteins.^117^

Furthermore, autophagy activates FAO to replenish TCA cycle intermediates, counteracting 5‐FU‐induced metabolic catastrophe.^118^ Clinically, 81% of 5‐FU‐resistant CRC tissues exhibit ATG5 K103la positivity, with autophagic flux (LC3‐II/p62 ratio) 3.2‐fold higher than in treatment‐sensitive counterparts. A clinical trial combining the autophagy inhibitor hydroxychloroquine with 5‐FU showed a disease control rate of 68%, compared with 32% in the control group, and the key signalling pathways and therapeutic implications of this resistance mechanism are also supplemented in Table 2.^119^

The Kla of autophagy proteins and subsequent 5‐FU resistance in CRC display significant spatial and clonal heterogeneity: (1) spatial heterogeneity: the hypoxic tumour core has constitutive Kla of ATG5 K103la, LC3B K49la and p62 K7la, leading to persistent high autophagic flux and intrinsic 5‐FU resistance; the tumour periphery has transient Kla of these autophagy proteins under 5‐FU treatment, and autophagic flux is moderate and reversible, making the periphery sensitive to the combination of hydroxychloroquine and 5‐FU.^99^, ^120^ (2) Clonal heterogeneity: ATG5 K103la, LC3B K49la and p62 K7la are stable clonal markers of 5‐FU‐resistant CRC, and the autophagic flux in these clones is constitutively high, which is a key mechanism for clearing 5‐FU‐induced toxic proteins; in contrast, 5‐FU‐sensitive clones only display transient Kla of autophagy proteins under acute metabolic stress, and autophagic flux can be effectively inhibited by 5‐FU.^4^ (3) CTC heterogeneity: CRC CTCs exhibit selective Kla of autophagy proteins (e.g., ATG14 K156la, ULK1 K750la) that is distinct from primary tumour cells, which enhances autophagosome formation under glucose limitation in the bloodstream and enables CTCs to utilise FAO for energy production, a critical survival mechanism for CTCs and a key basis for 5‐FU resistance in metastatic CRC.^114^

### 4.3 Lung cancer: EGFR‐TKI resistance

#### 4.3.1 H3K9 Kla‐driven EMT

Studies indicate that a high‐lactate microenvironment induces histone H3 lysine 9 Kla (H3K9la),^121^ triggering epigenetic reprogramming of EMT genes. For instance, at the E‐cadherin locus, H3K9la occupies the promoter and recruits DNMT3A to mediate methylation, thereby silencing transcription. This process leads to loss of cell adhesion and promotes tumour cell dissociation and invasion.^122^ At the Vimentin locus, H3K9la opens enhancer regions to strengthen ZEB1 binding affinity, up‐regulating expression and remodelling the cytoskeleton to enhance migratory capacity.^123^ At the SNAI1 locus, H3K9la competitively displaces H3K9me3 to activate transcription, which in turn suppresses E‐cadherin expression and acts as a core driver of EMT.^124^ EMT confers stem‐like properties to tumour cells, reducing EGFR‐TKI sensitivity and synchronously promoting metastasis and drug resistance.^125^ Clinically, H3K9la levels positively correlate with EMT scores in EGFR‐mutant lung cancer, with TKI‐treated patients exhibiting high H3K9la showing a median PFS of only 3.2 versus 12.1 months in controls, and the specific mechanism of H3K9la driving EMT is summarised in Table 2, illustrating the link between epigenetic reprogramming and drug resistance.^126^

H3K9la‐driven EMT and EGFR‐TKI resistance in lung cancer exhibit pronounced spatial and clonal heterogeneity, which is a key feature of intratumoural resistance diversity: (1) spatial heterogeneity: the hypoxic tumour core has extremely high H3K9la levels, leading to constitutive EMT activation (E‐cadherin silencing, Vimentin and SNAI1 up‐regulation) and stem‐like properties, forming intrinsic EGFR‐TKI resistance; the tumour periphery has moderate H3K9la levels, with partial EMT activation that is reversible by inhibiting Kla, making the periphery a potential target for EGFR‐TKI combined with Kla inhibitors. (2) Clonal heterogeneity: EGFR‐TKI‐resistant lung cancer clones possess H3K9la as a stable clonal marker, and the EMT gene locus in these clones has a unique chromatin landscape that is highly susceptible to H3K9la‐mediated epigenetic reprogramming; additionally, these clones exhibit a higher Kla level of stemness regulators (e.g., OCT4 K118la) than sensitive clones, further enhancing stem‐like properties and MDR. (3) CTC heterogeneity: lung cancer CTCs exhibit H3K9la levels that are significantly higher than primary tumour cells, leading to constitutive EMT activation and a mesenchymal phenotype, which enhances CTC motility and invasion in the bloodstream; additionally, CTCs exhibit Kla of SOX2 (K7la) that is distinct from primary tumour cells, sustaining stemness and enabling CTC colonisation at metastatic sites (e.g., brain, bone), a key mechanism for EGFR‐TKI‐resistant distant metastasis.

#### 4.3.2 Regulatory role of TFAM Kla in mitochondrial metabolism

In lung cancer, Kla at lysine 88 of the mitochondrial TF A (TFAM K88la) drives metabolic reprogramming through three functional alterations: (1) enhanced mtDNA transcription, where TFAM K88la strengthens D‐loop binding to increase mtDNA copy number and up‐regulate respiratory chain complexes I–IV expression^127^; (2) activated OXPHOS, boosting ATP production efficiency to compensate for TKI‐induced energy crisis and sustain cell survival, thereby perpetuating drug resistance; (3) up‐regulated antioxidant defences, where SOD2/GPx4 scavenge TKI‐generated ROS to inhibit ferroptosis and evade TKI cytotoxicity.^128^ This mechanism represents mitochondrial metabolic compensation, enabling tumour cells to circumvent EGFR‐TKI‐mediated metabolic suppression and develop acquired resistance.^45^ Clinically, 92% of TKI‐resistant lung cancer biopsy samples exhibit TFAM K88la positivity, and the correlation between modification levels and plasma lactate concentrations is presented in Table 2.^127^

TFAM K88la‐mediated mitochondrial metabolic compensation and EGFR‐TKI resistance in lung cancer display distinct intratumoural heterogeneity: (1) spatial heterogeneity: the hypoxic tumour core has constitutive TFAM K88la, leading to persistent OXPHOS activation and antioxidant defence up‐regulation, which effectively compensates for the energy crisis induced by EGFR‐TKI and forms intrinsic resistance; the tumour periphery has transient TFAM K88la under EGFR‐TKI treatment, and OXPHOS activation is moderate and reversible, making the periphery sensitive to EGFR‐TKI combined with OXPHOS inhibitors.^99^, ^125^ (2) Clonal heterogeneity: TFAM K88la is a stable clonal marker of EGFR‐TKI‐resistant lung cancer, and the mitochondrial metabolism of these clones is characterised by constitutive OXPHOS activation, which is not observed in EGFR‐TKI‐sensitive clones; additionally, resistant clones exhibit higher Kla levels of antioxidant proteins (SOD2 K68la, GPx4 K14la) than sensitive clones, further enhancing ferroptosis resistance.^129^ (3) CTC heterogeneity: lung cancer CTCs exhibit TFAM K88la levels that are twofold higher than primary tumour cells, leading to enhanced mtDNA transcription and OXPHOS activation, which provides sufficient ATP for CTC survival in the bloodstream; additionally, CTCs exhibit Kla of mitochondrial anti‐oxidative stress proteins (e.g., PRDX3 K9la) that is distinct from primary tumour cells, effectively scavenging ROS in the circulation and preventing CTC ferroptosis, a key survival mechanism for EGFR‐TKI‐resistant CTCs.^72^

### 4.4 Pancreatic cancer: immunotherapy resistance

#### 4.4.1 H3K122 Kla in CAFs and stromal barrier formation

Elevated lactate within the pancreatic TME induces histone H3 lysine 122 Kla (H3K122la) in cancer‐associated fibroblasts (CAFs),^130^ driving stromal barrier reinforcement through three mechanisms: (1) H3K122la induces chromatin opening at the promoters of COL1A1 and FN1, enhancing their transcription. This increases collagen and fibronectin deposition, thickening the physical barrier and significantly reducing T‐cell infiltration.^131^ (2) H3K122la activates the transcription of IL‐6 and IL‐8, recruiting myeloid‐derived suppressor cells (MDSCs) and Tregs to establish an immunosuppressive niche.^132^ (3) H3K122la suppresses LATS1, activating YAP1 to promote fibrogenic gene expression and increase stromal stiffness.^133^ This dense stromal barrier physically blocks immune cell infiltration, preventing anti‐PD‐1/L1 antibodies from contacting tumour cells and resulting in primary immunotherapy resistance^134^ (Table 2).

Pancreatic cancer is characterised by a dense stromal microenvironment, and H3K122la in CAFs‐driven stromal barrier formation exhibits extreme spatial heterogeneity and clonal specificity: (1) spatial heterogeneity: the hypoxic tumour core has the highest lactate levels, inducing hyper‐Kla of H3K122la in CAFs and forming an extremely dense stromal barrier with severe collagen/fibronectin deposition and MDSC/Treg infiltration, which is the primary site of primary immunotherapy resistance in pancreatic cancer; the tumour periphery has moderate lactate levels, with milder H3K122la in CAFs and a relatively loose stromal barrier, which has partial T cell infiltration and is a potential target for combination therapy with stromal‐disrupting agents and immunotherapy.^134^, ^135^ (2) Clonal heterogeneity: immunotherapy‐resistant pancreatic cancer clones drive constitutive H3K122la in adjacent CAFs through paracrine lactate secretion, and the CAFs in the clone microenvironment exhibit a unique ‘fibroblast‐immune suppressor’ phenotype driven by Kla, which is not observed in immunotherapy‐sensitive clones; additionally, resistant clones exhibit higher lactate secretion levels than sensitive clones, further enhancing CAF activation and stromal barrier formation.^136^, ^137^ (3) CTC–stroma interaction heterogeneity: pancreatic cancer CTCs secrete lactate to induce Kla of stromal fibroblasts in the circulatory system and metastatic sites (e.g., liver), leading to the formation of a pre‐metastatic niche with a loose stromal barrier and immunosuppression, which provides a favourable microenvironment for CTC colonisation and metastasis; this Kla‐mediated pre‐metastatic niche formation is a key mechanism for immunotherapy resistance in metastatic pancreatic cancer.^138^

#### 4.4.2 CXCR4 Kla‐mediated tumour cell migration and drug resistance

Kla of the chemokine receptor CXCR4 at lysine 288 (CXCR4 K288la) in pancreatic tumour cells exerts dual effects in promoting migration and drug resistance through three mechanisms: (1) enhanced receptor stability: CXCR4 K288la blocks ubiquitination, doubling the receptor half‐life and enabling sustained activation of the CXCL12/CXCR4 axis to drive metastasis.^139^ (2) Amplified downstream signalling: increased ERK/FAK phosphorylation enhances cellular motility, facilitating tumour cell escape to immune‐privileged sites (e.g., liver).^130^ (3) NF‐κB activation: this up‐regulates PD‐L1 expression, inducing local immune evasion.^132^ Collectively, this mechanism drives tumour cell migration to immune‐protected niches coupled with localised PD‐L1 up‐regulation, ultimately preventing immunotherapy‐mediated clearance of metastatic lesions and culminating in acquired resistance and recurrence (Table 2).

CXCR4 K288la‐mediated migration and immunotherapy resistance in pancreatic cancer exhibit distinct spatial and clonal heterogeneity, which is a key driver of tumour metastasis and acquired resistance: (1) spatial heterogeneity: the tumour periphery has higher CXCR4 K288la levels than the core, leading to enhanced motility and invasion of peripheral tumour cells, which is the primary source of CTCs and distant metastasis in pancreatic cancer^140^; the periphery also exhibits PD‐L1 up‐regulation driven by CXCR4 K288la/NF‐κB, forming local immune evasion and acquired immunotherapy resistance.^141^, ^142^ (2) Clonal heterogeneity: CXCR4 K288la is a stable clonal marker of immunotherapy‐resistant pancreatic cancer, and these clones exhibit constitutive activation of the CXCL12/CXCR4 axis and ERK/FAK signalling, leading to enhanced migration and invasion; additionally, resistant clones exhibit higher PD‐L1 expression levels than sensitive clones due to NF‐κB activation, further enhancing local immune evasion.^46^, ^143^ (3) CTC heterogeneity: pancreatic cancer CTCs exhibit CXCR4 K288la levels that are threefold higher than primary tumour cells, leading to constitutive activation of the CXCL12/CXCR4 axis and enhanced motility in the bloodstream^144^; additionally, CTCs exhibit Kla of CXCR7 (K198la) that is distinct from primary tumour cells, which synergises with CXCR4 K288la to promote CTC homing to immune‐privileged metastatic sites (e.g., liver, peritoneum), a key mechanism for immunotherapy resistance in metastatic pancreatic cancer.^103^, ^134^, ^144^

## 5 TARGETING Kla THERAPEUTIC STRATEGIES

Based on the universal molecular mechanisms of Kla‐mediated tumour drug resistance and the tumour‐specific and intratumoural spatial/clonal heterogeneity of lactylation elaborated in Sections 3 and 4, this section will explore potential Kla‐targeted therapeutic strategies, including inhibition of key lactate‐metabolising enzymes, modulation of Kla‐modifying enzyme activity and combination therapies.^14^, ^16^ Notably, the design of Kla‐targeted therapeutic strategies must fully consider the intratumoural spatial and clonal heterogeneity of Kla: a single targeted therapy cannot effectively eliminate all tumour cell populations (core, periphery, CTCs, drug‐resistant clones) with distinct Kla profiles, and personalised and combinatorial therapeutic strategies based on intratumoural Kla heterogeneity are the key to improving the efficacy of Kla‐targeted therapy.^2^ This section will also discuss the current challenges and future directions of Kla‐targeted therapy, with a focus on addressing the bottlenecks caused by intratumoural Kla heterogeneity.^145^

### 5.1 Targeting key enzymes in lactate metabolism

#### 5.1.1 Research progress and clinical potential of LDHA inhibitors

LDHA is the key enzyme catalysing the conversion of pyruvate to lactate during glycolysis. Inhibiting LDHA can disrupt the Warburg effect in cancer cells, reduce lactate production and reverse TME acidification.^130^ The representative drug FX‐866 (currently in phase II clinical trials) is the first oral LDHA inhibitor, demonstrating significant tumour growth inhibition in melanoma and pancreatic cancer models. However, it exhibits dose‐limiting toxicities, such as metabolic acidosis.^146^ GSK2837808A is a highly selective inhibitor, with preclinical studies showing efficacy against triple‐negative breast cancer metastasis, but its development is limited by low bioavailability. Allosteric inhibitors (e.g., NHI‐1), which target the tetramer interface of LDHA, possess higher specificity. When combined with PD‐1/CTLA‐4 inhibitors, they can enhance the efficacy of immunotherapy.^147^ Nevertheless, it is crucial to note that tumour cells have substantial metabolic plasticity. Upon LDHA inhibition, they may activate compensatory metabolic pathways (including enhanced glutaminolysis, FAO or utilisation of alternative carbon sources) to re‐establish metabolic balance, leading to the emergence of drug‐resistant phenotypes and thus compromising the long‐term efficacy of LDHA inhibitor monotherapy. Therefore, it is imperative to explore multi‐target combination strategies. For instance, combining LDHA inhibitors with inhibitors of other metabolic pathways (e.g., glutaminase inhibitors) or immune checkpoint inhibitors can effectively block compensatory metabolic reprogramming, thereby enhancing therapeutic efficacy and delaying the onset of drug resistance (Table 3).^148^

**TABLE 3:** Summary of inhibitors targeting lactate metabolism.

Target enzyme	Representative agent(s)	Mechanism of action
LDHA	FX‐866	Inhibits LDHA, thereby blocking the Warburg effect, reducing lactate production and reversing tumour microenvironment (TME) acidification
	GSK2837808A	Highly selective inhibitor of LDHA
	NHI‐1 (allosteric inhibitor)	Specifically inhibits LDHA by binding to its tetramer interface; enhances immunotherapy efficacy when combined with PD‐1/CTLA‐4 inhibitors
MCT1/4	AZD3965	Selectively inhibits MCT1, blocking lactate efflux, leading to intracellular lactate accumulation and pH dysregulation; synergises with radiotherapy and anti‐angiogenic agents
PDHK	DCA (dichloroacetate)	Inhibits PDHK, activating pyruvate dehydrogenase (PDH) to promote pyruvate oxidation in mitochondria, thereby reducing lactate generation; favourable safety profile in glioma clinical trials
HIF‐1α	PX‐478	Inhibits HIF‐1α, down‐regulating the expression of LDHA and MCT4; effective for hypoxic solid tumours
GPR81 (lactate receptor)	– (Antagonist)	Blocks lactate‐mediated immunosuppressive signalling cascades, enhances T‐cell infiltration and remodels the immune microenvironment

*Note*: ‘–’ indicates that specific agent names were not provided in the source material.

Considering the intratumoural spatial heterogeneity of lactate production (the hypoxic tumour core has the highest LDHA activity and lactate production, while the periphery has moderate LDHA activity, and CTCs exhibit LDHA isoform switching and activity adaptation to circulatory nutrient limitation), the administration of LDHA inhibitors should adopt a ‘gradient dosing + isoform‐specific inhibition’ strategy based on the lactate concentration and LDHA molecular characteristics in different tumour regions and cell populations^16^, ^149^: higher doses of pan‐LDHA inhibitors for the tumour core to effectively inhibit hyper‐lactate production and break the ‘hypoxia–glycolysis–Kla’ positive feedback loop^103^, ^150^; moderate doses of LDHA inhibitors combined with glycolytic flux modulators for the tumour periphery to avoid excessive metabolic stress and compensatory pathway activation while targeting Kla associated with cell invasion^151^, ^152^; and isoform‐specific LDHA inhibitors (targeting LDHA2, the dominant isoform in CTCs) for CTCs to reduce CTC lactate secretion and inhibit Kla‐mediated DNA damage repair and stemness maintenance in the bloodstream.^153^, ^154^ For drug‐resistant clones with constitutively high LDHA activity and unique Kla signatures (e.g., LDHA K56la), LDHA inhibitors should be combined with clonal‐specific targeted therapies (e.g., inhibitors of the Kla writer proteins specifically activated in resistant clones) to block both lactate production and Kla‐mediated resistance mechanisms,^46^ and co‐target the metabolic compensation pathways uniquely activated in these clones (e.g., enhanced glutaminolysis).^155^

#### 5.1.2 Targeting strategies for other lactate metabolism‐related enzymes

In addition to LDHA, other key molecules involved in lactate metabolism have emerged as promising therapeutic targets, with their inhibitors exhibiting diverse mechanisms of action and clinical potential (Table 3).

1. MCTs (MCT1/4) mediate lactate efflux across cell membranes, and their inhibition represents a critical strategy to disrupt lactate homeostasis in the TME. AZD3965, a prototypical MCT1‐selective inhibitor currently in phase I/II clinical trials, blocks lactate export, leading to intracellular lactate accumulation and pH dysregulation.^156^ While demonstrating significant efficacy in diffuse large B‐cell lymphoma, its clinical application is limited by retinal toxicity. Notably, combination regimens involving AZD3965 have shown enhanced therapeutic benefits: synergistic effects with radiotherapy promote glioma cell apoptosis, and co‐administration with anti‐angiogenic agents (e.g., bevacizumab) inhibits tumour vascular normalisation.^157^
2. Pyruvate dehydrogenase kinase (PDHK) inhibitors, such as dichloroacetate (DCA), reduce lactate production by activating pyruvate dehydrogenase (PDH), thereby promoting pyruvate entry into mitochondrial oxidative metabolism. DCA has demonstrated favourable safety profiles in glioma clinical trials, supporting its potential for further translational development.^158^
3. HIF‐1α indirectly regulates lactate metabolism by transcriptionally up‐regulating LDHA and MCT4. Inhibitors like PX‐478 suppress HIF‐1α activity, down‐regulating these effector molecules and thus representing a viable strategy for hypoxic solid tumours.^159^
4. Lactate receptor (GPR81) antagonists, currently in preclinical development, block lactate‐mediated immunosuppressive signalling cascades and enhance T‐cell infiltration, offering a novel approach to remodel the immune microenvironment.^160^

For these lactate metabolism‐related enzymes, their targeting strategies must also consider intratumoural Kla heterogeneity: (1) MCT4 is highly expressed in the hypoxic tumour core and drug‐resistant clones, while MCT1 is predominantly expressed in the tumour periphery and CTCs, so MCT1/4 inhibitors should be selected based on tumour region and cell population (e.g., MCT4 inhibitors for the core and drug‐resistant clones, MCT1 inhibitors for the periphery and CTCs).^161^, ^162^, ^163^ (2) HIF‐1α is constitutively activated in the tumour core and drug‐resistant clones, so HIF‐1α inhibitors (e.g., PX‐478) are particularly effective for these cell populations, and combination with LDHA inhibitors can form a ‘double blockade’ of lactate production.^164^, ^165^, ^166^, ^167^ (3) GPR81 is highly expressed in TAMs and monocytes in the tumour core and drug‐resistant clone microenvironments, so GPR81 antagonists are a key strategy to reverse Kla‐mediated immunosuppression in these regions, and combination with PD‐1 inhibitors can significantly enhance immunotherapy efficacy.^168^, ^169^, ^170^

Future research should prioritise the development of multi‐target inhibitors and sequential dosing strategies to overcome adaptive resistance. For microenvironment modulation, combining these agents with immune checkpoint inhibitors (e.g., anti‐PD‐L1) may reverse lactate‐induced T‐cell dysfunction.^14^ Additionally, dynamic monitoring of lactate metabolism via PET‐CT (using 18F‐FDG in combination with 11C‐lactate) holds promise as a biomarker strategy to guide precision therapeutic interventions.^171^ Importantly, the development of lactate metabolism imaging technology should be further advanced to realise real‐time monitoring of lactate concentration and Kla levels in different tumour regions (core, periphery) and cell populations (CTCs, drug‐resistant clones), which is the key to guiding personalised Kla‐targeted therapy based on intratumoural heterogeneity.^149^, ^172^, ^173^, ^174^

### 5.2 Modulating the activity of Kla‐modifying enzymes

The established classical writer–eraser–reader regulatory paradigm of Kla, and the key targets of modulating Kla‐modifying enzyme activity are the writers (lactyltransferases such as p300/CBP) and erasers (delactylases such as SIRT family and HDAC1–3) in this framework.^145^, ^175^ Targeting the catalytic activity of lactyltransferases or activating the delactylase activity of erasers can directly regulate the intracellular Kla level, thus reversing the Kla‐driven tumour drug resistance phenotype.^16^ Notably, the writer–eraser–reader regulatory framework of Kla exhibits distinct activation patterns in different tumour regions and cell populations (core, periphery, CTCs, drug‐resistant clones), so the modulation of Kla‐modifying enzyme activity must adopt a ‘targeted regulation’ strategy based on the specific activation status of the framework in different cell populations, rather than global activation or inhibition.^2^, ^11^

Kla, a PTM that regulates gene expression through the covalent binding of lactyl groups to histone lysine residues, is primarily mediated by the histone acetyltransferases p300 and CBP. The core regulatory mechanism involves the activation of p300/CBP by lactate in the TME, which in turn catalyses histone modifications such as H3K18la and H3K9la. These modifications promote the transcription of immune evasion‐related genes (e.g., PD‐L1) and pro‐oncogenic genes, thereby contributing to tumour progression.^156^ Notably, recent studies have expanded the regulatory scope of p300/CBP‐mediated Kla to non‐histone apoptotic regulators: p300 directly catalyses Kla of the anti‐apoptotic protein BCL‐XL at K122, stabilising its interaction with pro‐apoptotic BAX and blocking MOMP in lung and gastric cancers.^176^ Additionally, p300/CBP‐driven H3K18la directly silences the pro‐apoptotic gene BIM by recruiting the transcriptional repressor DNMT3A, further reinforcing apoptotic resistance in CBP‐deficient tumours.^176^ In sepsis‐associated acute lung injury, p300‐mediated H3K18la up‐regulates METTL3, which promotes ACSL4‐dependent ferroptosis (a form of regulated cell death closely linked to apoptotic signalling) in alveolar epithelial cells, highlighting Kla's crosstalk with multiple cell death pathways.^177^

Spatially and clonally, p300/CBP exhibits distinct activation patterns: (1) p300/CBP is constitutively activated in the hypoxic tumour core and drug‐resistant clones, mediating global histone Kla and non‐histone Kla of anti‐apoptotic/immune escape proteins, so p300/CBP inhibitors (e.g., A485) are particularly effective for these cell populations.^1^, ^5^ (2) KAT2A is preferentially activated in the tumour periphery, catalysing Kla of invasion/metastasis‐related genes, so KAT2A inhibitors are a key strategy to target the periphery and inhibit tumour metastasis.^178^ (3) AARS1/AARS2 is highly expressed in CTCs, mediating Kla of DNA damage repair and stemness proteins, so AARS1/AARS2 inhibitors are a novel target to inhibit CTC survival and metastasis.^179^ For erasers, SIRT2 is significantly inhibited in the tumour core and drug‐resistant clones, leading to Kla accumulation, so SIRT2 activators are effective for these cell populations; SIRT3 is the dominant delactylase in CTCs, so SIRT3 activation can specifically reduce CTC Kla levels and inhibit CTC survival.^2^, ^180^

A485, the first selective inhibitor targeting the catalytic domain of p300/CBP, has been shown in preclinical studies to significantly reduce Kla levels in tumour cells and inhibit the growth of triple‐negative breast cancer.^181^ Recent preclinical data demonstrate that A485 alone does not induce substantial apoptosis but acts as a potent sensitiser for tumour necrosis factor‐related apoptosis‐inducing ligand (TRAIL): in both EGFR‐TKI‐sensitive and resistant non‐small‐cell lung cancer (NSCLC) cells, the combination of A485 and TRAIL synergistically increases apoptotic cell death by 2.3‐fold and inhibits 3D spheroid growth, which is associated with reduced H3K18la and restored BIM expression.^182^ A485 also exhibits lineage‐specific efficacy in androgen receptor‐positive prostate cancer, where it suppresses the AR transcriptional program and induces apoptosis in castration‐resistant xenograft models.^183^ Additionally, PROTAC degraders (e.g., dCBP‐1, currently in preclinical development) block Kla signalling by inducing ubiquitin‐mediated degradation of p300/CBP proteins, which may overcome the resistance associated with traditional inhibitors.^184^ A novel oral p300/CBP PROTAC, CBPD‐409 selectively degrades p300/CBP in AR‐positive prostate cancer cells (with minimal effects on normal cells) and eliminates H2B N‐terminal acetylation (H2BNTac) and H3K27ac signals, leading to MYC down‐regulation and apoptotic cell death, its efficacy is far superior to bromodomain inhibitors and HAT inhibitors, and it synergises with AR antagonists to suppress tumour growth. Notably, combining these PROTAC degraders with PD‐1 inhibitors can reverse Kla‐induced T‐cell exhaustion.^185^ Mechanistically, this combination not only reduces tumour cell Kla but also mitigates Kla of T‐cell TF NFATc1, restoring IL‐2 production and T‐cell‐mediated cytotoxicity against tumour cells.^19^ However, a critical challenge remains: since p300/CBP regulates the expression of essential genes in normal cells, achieving tissue‐specific delivery of these inhibitors is imperative to minimise off‐target effects. CBPD‐409 addresses this in part through its inherent selectivity for AR‐positive prostate cancer cells, as demonstrated by minimal toxicity in long‐term preclinical dosing.^185^

To further improve the efficacy of lactyltransferase inhibitors and reduce off‐target effects, the development of tumour region/cell population‐specific delivery systems is crucial: (1) for the hypoxic tumour core, develop hypoxia‐responsive p300/CBP inhibitor prodrugs that are only activated in the hypoxic microenvironment, avoiding inhibition of p300/CBP in normal cells.^186^, ^187^ (2) For drug‐resistant clones, develop PROTAC degraders targeting the unique Kla signature of resistant clones (e.g., p300/CBP degraders linked to clone‐specific surface markers), realising selective degradation of p300/CBP in resistant clones.^188^, ^189^ (3) For CTCs, develop lipid nanoparticle‐encapsulated AARS1/AARS2 inhibitors that target CTCs, specifically reducing CTC Kla levels without affecting normal blood cells.^190^, ^191^, ^192^

Conversely, the delactylase SIRT2 primarily exerts tumour‐suppressive effects by removing histone Kla marks. Activation of SIRT2 reduces H3K18la levels, restores the expression of tumour suppressor genes (e.g., p53) and inhibits key enzymes involved in the Warburg effect (e.g., HK2, PFKFB3).^193^ Beyond SIRT2, SIRT5 (a NAD+‐dependent sirtuin) has emerged as a key delactylase regulating apoptosis: in SH‐EP neuroblastoma cells, SIRT5 overexpression protects against staurosporine‐induced apoptosis by reducing oxidative stress, while SIRT5 knockdown increases caspase 3/7 activity and apoptotic cell numbers.^194^ In cardiomyocytes, SIRT5 interacts directly with BCL‐XL to maintain its anti‐apoptotic function, and SIRT5 down‐regulation under oxidative stress exacerbates apoptotic cell death.^195^ These findings highlight the context‐dependent role of sirtuin family delactylases in apoptotic regulation, with SIRT5 exerting anti‐apoptotic effects in certain cell types (distinct from SIRT2's tumour‐suppressive role in most cancers).^19^ It is worth noting that SIRT2 may exert pro‐tumourigenic functions in certain cancer types (e.g., breast cancer),^196^ highlighting the need for developing biomarkers (such as lactylomics) to enable precise patient stratification.^197^ Recent comprehensive analyses identify Kla‐related genes (e.g., DDX39A, ARID3A) as potential diagnostic biomarkers for diseases ranging from atrial fibrillation to cancer, supporting the development of lactylome‐based stratification tools.^198^ Additionally, the KDAC family (major delactylases) and TRIM33 (a Kla‐specific ‘reader’ protein) form a complex regulatory network that modulates apoptotic signalling by fine‐tuning Kla levels of both histone and non‐histone substrates.^19^

The activation of delactylases should also adopt a cell population‐specific strategy based on intratumoural Kla heterogeneity: (1) SIRT2 activators are the primary choice for the hypoxic tumour core and drug‐resistant clones, where SIRT2 is significantly inhibited, to restore delactylase activity and reduce Kla accumulation^9^. (2) SIRT3 activators are specifically used for CTCs, where SIRT3 is the dominant delactylase, to reduce CTC Kla levels and inhibit CTC survival in the bloodstream.^179^, ^180^ (3) HDAC1–3 activators are effective for the tumour periphery, where HDAC1–3 retains partial activity, to further enhance delactylase activity and reverse inducible Kla‐mediated resistance. For the context‐dependent role of sirtuins, lactylome‐based patient stratification is essential to select appropriate delactylase modulators for different cancer types and tumour cell populations.^2^, ^9^, ^41^, ^48^

A promising therapeutic strategy involves combining p300 inhibitors with SIRT2 activators, which simultaneously block Kla generation and accelerate its clearance. In pancreatic cancer models, this combination has demonstrated a threefold increase in efficacy compared with monotherapy.^159^ Expanding on this, the triple combination of A485 (p300 inhibitor), a SIRT2 activator, and TRAIL has shown superior apoptotic induction in EGFR‐TKI‐resistant NSCLC cells, with a 40% higher cell death rate than dual combinations.^182^ In AR‐positive prostate cancer, combining CBPD‐409 (p300/CBP PROTAC) with a SIRT2 activator enhances MYC down‐regulation and apoptotic sensitivity, achieving tumour regression in 60% of preclinical models without significant systemic toxicity^185^. Currently, the clinical translation of such strategies is hindered by challenges, including the lack of mature real‐time Kla monitoring techniques and the need to ensure the long‐term safety of epigenetic modulation. Notably, the emerging understanding of Kla's ‘writer–eraser–reader’ network (e.g., p300/CBP as writers, KDACs/SIRTs as erasers, TRIM33 as a reader) provides novel therapeutic targets with higher specificity than traditional metabolic inhibitors, reducing off‐target risks Additionally, lactylome‐based liquid biopsy (detecting circulating lactylated BCL‐XL/BIM) is being validated as a predictive biomarker for patient response to p300/CBP‐targeted therapies,^198^ addressing the monitoring gap.

### 5.3 Target gene knockout verification

To establish the causal link between core Kla‑related targets, Kla modification and tumour drug resistance, CRISPR/Cas9‑mediated gene knockout (KO) and conditional KO animal models have been widely applied to validate functional specificity. Below are key findings from six representative PubMed‑indexed studies focusing on LDHA, p300 (EP300), SIRT2 and NBS1.

#### 5.3.1 LDHA KO abolishes lactate supply and global Kla to reverse chemoresistance

LDHA is the rate‑limiting enzyme for lactate production. LDHA‑KO directly blocks lactate supply and eliminates the substrate basis for Kla. In cisplatin‑resistant gastric cancer cells, LDHA‑KO reduced lactate production by >70% and almost eliminated global Kla (Pan‑Kla) and NBS1 K388la. HR efficiency decreased by ∼60%, and cisplatin IC50 dropped by 6.2‑fold. In mouse xenografts, LDHA‑KO reduced H3K18la and H3K9la levels, inhibited tumour growth by ∼75%, and increased cisplatin response rate from 18 to 72%. These results confirm that LDHA is necessary for Kla and Kla‑driven chemoresistance.^11^, ^154^

#### 5.3.2 p300 (EP300) KO inhibits histone Kla and sensitises tumours to therapy

p300 acts as a major lactyltransferase writer for histone Kla. In HCT116 and HEK293T cells, EP300‑KO reduced global histone Kla by ∼55%, with H3K18la and H4K12la decreased by ∼70%. In colorectal CSCs, p300‑KO down‐regulated H4K12la and GCLC expression, restoring oxaliplatin sensitivity. In EGFR‑mutant lung cancer, p300‑KO reduced H3K9la, reversed EMT and restored EGFR‑TKI sensitivity. p300 is therefore required for histone Kla and epigenetic reprogramming in drug resistance.^49^, ^183^

#### 5.3.3 SIRT2 KO enhances Kla accumulation and exacerbates drug resistance

SIRT2 functions as a key delactylase eraser that removes Kla marks. In breast and gastric cancer cells, SIRT2‑KO increased H3K18la and H3K9la by ∼2.8‑fold, stabilised BCL‑2 and MCL‑1 and reduced apoptosis by ∼65%. Resistance to paclitaxel and trastuzumab increased by 4.5‑fold. Mechanistically, SIRT2‑KO removes the ‘brake’ on Kla, leading to hyper‑Kla and strengthened anti‑apoptotic signalling. SIRT2 negatively regulates Kla and drug resistance, and its inactivation is sufficient to drive resistance.^36^

#### 5.3.4 NBS1 KO or K388R mutation blocks Kla‑dependent DNA repair and resistance

NBS1 K388 is a critical Kla site that promotes DNA DSB repair. NBS1‑KO or K388R Kla‑null mutation abolished Kla at this site and impaired MRN complex assembly. HR efficiency was reduced by ∼70%, and lactate failed to induce cisplatin or radiation resistance. LDHA overexpression could not rescue resistance in NBS1 K388R cells, demonstrating that drug resistance strictly depends on NBS1 Kla.

NBS1 K388 Kla is necessary and sufficient for lactate‑mediated DNA repair and therapeutic resistance.

### 5.4 Combination therapy strategies

**FIGURE 5:**
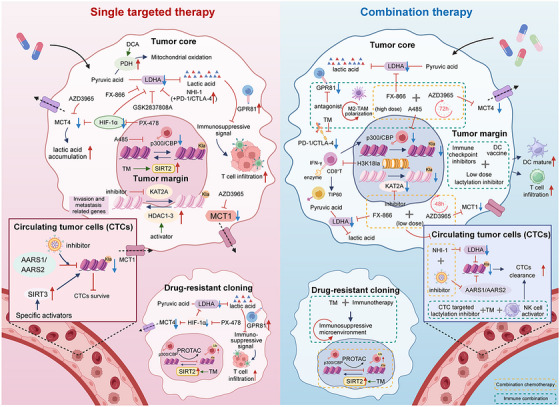
Synergistic mechanisms of lysine lactylation (Kla)‐targeted drugs and traditional therapies.

The design of Kla‐targeted combination therapies must be guided by the intratumoural spatial and clonal heterogeneity of Kla, with the core principle of matching therapeutic regimens to the Kla characteristics of different tumour cell populations, that is, adopting distinct combination strategies for the tumour core, periphery, CTCs and drug‐resistant clones, and integrating regional/clonal‐specific targeted therapy with conventional chemotherapy, immunotherapy and anti‐metastasis therapy to achieve comprehensive elimination of all tumour cell populations with divergent Kla profiles.^2^, ^48^, ^190^, ^199^, ^200^ The synergistic mechanisms of Kla‐targeted drugs combined with conventional therapies are illustrated in Figure 5. Moreover, the timing and sequence of drug administration should be optimised based on the dynamic changes of Kla in different cell populations under therapeutic pressure, such as lactate reduction first in the core, targeted inhibition later in the periphery and continuous clearance of CTCs throughout the process.^5^, ^201^, ^202^

**FIGURE 6:**
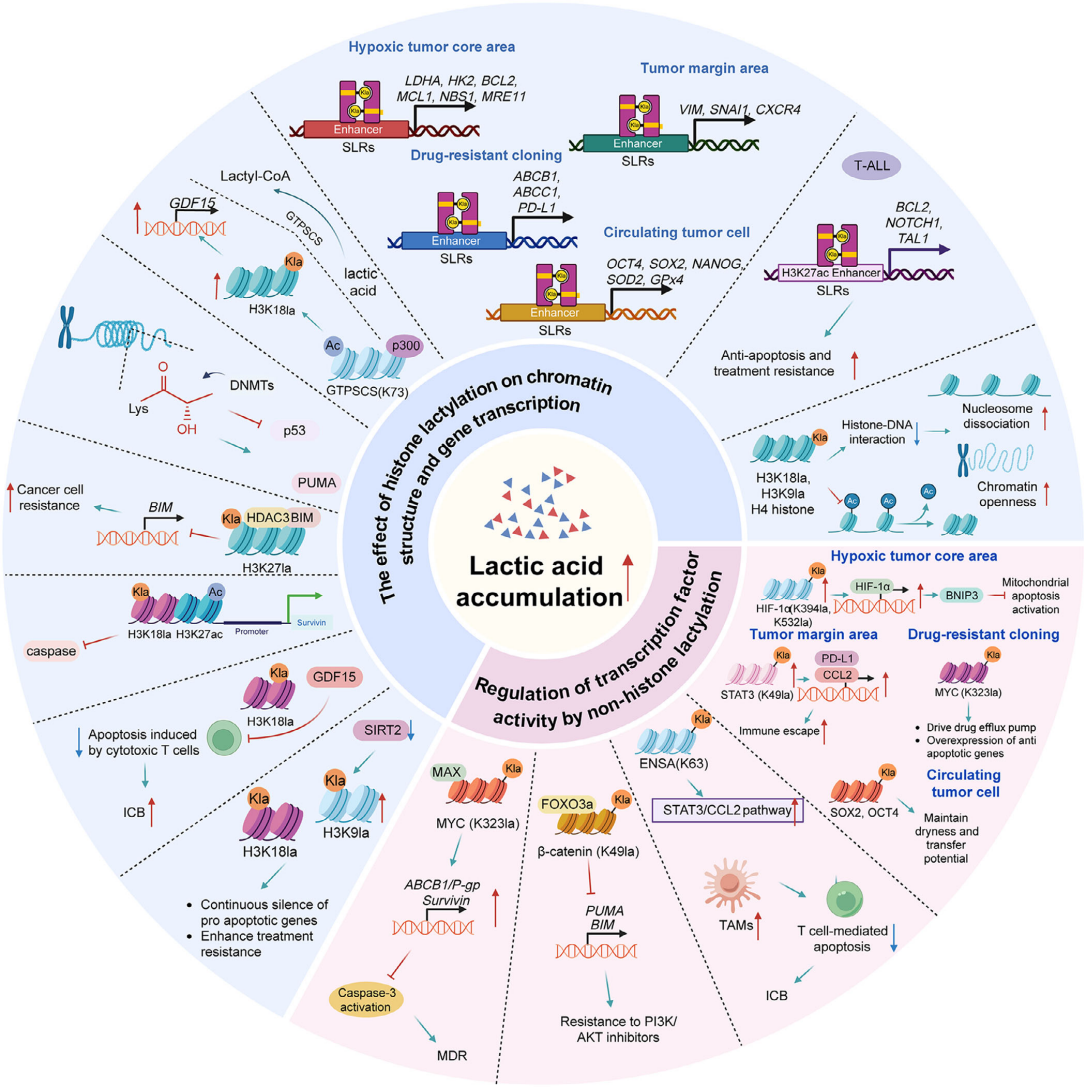
Epigenetic reprogramming and transcriptional regulation by histone and non‐histone lysine lactylation (Kla).

In the combined application of Kla‐targeting agents with conventional chemotherapy, the high lactate levels within the TME can reduce intracellular concentrations of chemotherapeutic drugs by activating HIF‐1α and MRP1.^203^ Conversely, inhibiting Kla modification can sensitise tumour cells to chemotherapy. A promising therapeutic regimen adopts a ‘lactate reduction first, chemotherapy second’ strategy: preclinical studies have shown that pretreatment with LDHA inhibitors (e.g., FX‐866) or MCT1 inhibitors (AZD3965) for 48 h to lower the pH of the TME, followed by administration of chemotherapeutic agents, results in a 2.3‐fold extension of survival in pancreatic cancer models.^204^ Based on intratumoural Kla heterogeneity, this sequential strategy should be upgraded to a spatial gradient sequential therapy: for the hypoxic tumour core (with the highest lactate levels and intrinsic chemotherapy resistance), pretreat with high‐dose LDHA/MCT4 inhibitors and p300/CBP inhibitors for 72 h to fully reduce lactate production and Kla levels, then administer chemotherapy to enhance drug penetration and cytotoxicity^2^; for the tumour periphery (with moderate lactate levels and inducible chemotherapy resistance), pretreat with low‐dose LDHA/MCT1 inhibitors and KAT2A blockers for 48 h, then combine chemotherapy with anti‐invasion agents to prevent peripheral tumour cells from escaping as CTCs; for drug‐resistant clones (with clonal‐specific Kla signatures and MDR), combine chemotherapy with clonal‐specific PROTAC degraders and delactylase activators to target the unique Kla‐mediated resistance mechanisms of these clones^205^; for CTCs (the main source of distant metastasis), administer isoform‐specific LDHA inhibitors and AARS1/AARS2 inhibitors throughout the chemotherapy process to continuously reduce CTC Kla levels and enhance the clearance of CTCs by chemotherapy and the immune system^7^. Low‐dose administration and optimised 48/72 h pretreatment scheduling are rationally adopted to avoid abrupt metabolic stress and compensatory pathway activation, while maximising the therapeutic synergy of combination regimens. Additionally, efficacy can be achieved by simultaneously blocking metabolic compensation mechanisms. For example, combining SIRT2 activators (TM) with paclitaxel inhibits the shift from glycolysis to OXPHOS, thereby overcoming drug resistance in breast cancer.^196^ For the tumour core and drug‐resistant clones that are prone to metabolic compensation after Kla inhibition, combine Kla‐targeting agents with inhibitors of compensatory metabolic pathways (e.g., glutaminase inhibitors, FAO inhibitors) to block the metabolic adaptation of tumour cells and avoid the emergence of secondary drug resistance.^1^, ^14^ However, strict monitoring of serum lactate levels is required during such combinations to prevent metabolic acidosis caused by the combined effects of chemotherapy and Kla inhibition (Figure 6).

The core breakthrough in synergising Kla‐targeted interventions with immunotherapy lies in reversing T‐cell suppression and remodelling the immune microenvironment. Specifically, Kla modifications (e.g., H3K18la) directly suppress IFN‐γ and granzyme B expression in CD8^+^ T cells, while targeting p300/CBP restores T‐cell cytotoxic function.^156^ SIRT2 activators reduce PD‐L1 Kla levels in tumour cells, reversing resistance to immune checkpoint inhibitors^206^; inhibiting Kla decreases Treg infiltration while enhancing dendritic cell maturation^207^ and blocking the lactate receptor GPR81 inhibits macrophage polarisation towards the M2 phenotype (Figure 6).^208^ Based on the intratumoural spatial and clonal heterogeneity of Kla‐mediated immunosuppression, the Kla‐targeted immunotherapy combination strategy should be designed to reverse immunosuppression in different tumour regions and cell populations in a targeted manner: the hypoxic tumour core is the main site of constitutive Kla‐mediated immunosuppression (hyper‐Kla of PD‐L1, M2‐TAM polarisation, exhausted CD8^+^ T cells), and thus combine PD‐1/CTLA‐4 inhibitors with LDHA/MCT4 inhibitors and GPR81 antagonists and p300/CBP inhibitors to fully reduce lactate levels, reverse M2‐TAM polarisation and down‐regulate Kla‐mediated PD‐L1 expression^1^, ^14^, ^209^; the tumour periphery has inducible immunosuppression under therapeutic pressure, and combine immune checkpoint inhibitors with low‐dose Kla inhibitors and dendritic cell vaccines to enhance DC maturation and T cell infiltration^210^; drug‐resistant clones have clonal‐specific Kla‐mediated immune escape (e.g., Foxp3 K268la in Tregs), and combine immunotherapy with clonal‐specific delactylase activators to reverse the immunosuppressive microenvironment of the clones^211^, ^212^; CTCs achieve immune escape in the circulation through Kla‐mediated PD‐L1 expression and myeloid cell suppression, and combine anti‐PD‐L1 antibodies with CTC‐targeted Kla inhibitors and NK cell activators to enhance immune clearance of CTCs and prevent distant metastasis.^14^, ^213^

Current optimal combination regimens include PROTAC degraders (e.g., dCBP‐1) co‐administered with anti‐PD‐1 antibodies, preclinical studies demonstrate this regimen increases T‐cell infiltration eightfold in immunologically cold tumours (e.g., prostate cancer) and achieves an objective response rate (ORR) of 52%, significantly higher than the 12% observed with anti‐PD‐1 monotherapy.^134^ Based on intratumoural Kla heterogeneity, this regimen can be further optimised into a ‘triple combination of Kla writer PROTAC and immune checkpoint inhibitor and eraser activator’, and adjusted according to different tumour cell populations^214^: for the tumour core, use p300/CBP PROTAC and anti‐PD‐1 and SIRT2 activator^134^; for the tumour periphery, use KAT2A PROTAC and anti‐CTLA‐4 and HDAC3 activator; for drug‐resistant clones, use clonal‐specific writer PROTAC and anti‐LAG‐3 and delactylase activator^103^; this optimised regimen can simultaneously block Kla generation, accelerate Kla clearance and reverse immune suppression, achieving a synergistic enhancement of immunotherapy efficacy in all tumour cell populations.^14^ Furthermore, combining SIRT2 activators (TM) with CAR‐T cell therapy enhances CAR‐T persistence by reducing tumour Kla levels, extending overall survival by 300% in glioblastoma mouse models.^215^ For solid tumours with dense stroma and low CAR‐T infiltration, this combination should be further modified by adding Kla inhibitors that target the tumour stroma (e.g., CAF‐specific H3K122la inhibitors) to reduce stromal barrier formation and enhance CAR‐T cell penetration into the tumour core^216^, ^217^; for CTCs and metastatic lesions, develop CAR‐T cells targeting CTC‐specific Kla markers to achieve specific recognition and killing of circulating and metastatic tumour cells.^218^ Regarding toxicity management, the potential increased risk of immune‐related adverse events (irAEs) necessitates careful dose adjustment and highlights the need for developing tumour‐targeted delivery systems to mitigate systemic toxicity.

In addition to chemotherapy and immunotherapy, Kla‐targeted combination therapies should also integrate anti‐metastasis therapy to target CTCs and metastatic tumour cells with unique Kla profiles.^16^ The core strategy is continuous inhibition of CTC Kla and blocking of pre‐metastatic niche formation: administer CTC‐specific Kla inhibitors (AARS1/AARS2 inhibitors, SIRT3 activators) throughout the treatment process to reduce CTC survival and stemness in the bloodstream^1^, ^7^, ^219^; combine with inhibitors of Kla‐mediated pre‐metastatic niche formation (e.g., CXCR4 K288la inhibitors, CAF Kla inhibitors) to block the interaction between CTCs and stromal cells in metastatic sites, and inhibit Kla‐mediated immunosuppression of the pre‐metastatic niche; for established metastatic lesions, adopt the same Kla‐targeted combination strategy as the primary tumour, matching the therapeutic regimen to the Kla characteristics of metastatic tumour cells (which are highly consistent with CTCs). This multi‐dimensional combination strategy can achieve comprehensive treatment of the primary tumour, CTCs and metastatic lesions, and fundamentally solve the problem of Kla‐mediated tumour metastasis and acquired drug resistance.^101^

In addition to the conventional well‐studied hot targets including LDHA, MCT1/4, p300/CBP and SIRT2 summarised above, this review further highlights a panel of truly novel and underexplored Kla‐related therapeutic targets that have not been fully reported in previous studies, which fills the gap of insufficient innovative target mining in current Kla research. These emerging novel targets mainly include three categories: (1) unreported specific Kla reader proteins represented by DPF2 and BRD4, which can specifically recognise unique histone Kla marks and drive oncogenic and drug‐resistance transcriptional programs independent of classical TRIM33/DNMT33; (2) multiple tumour‐specific unreported Kla modification sites such as LSD1 K503la, Tufm K286la and CXCR7 K198la, which are uniquely enriched in drug‐resistant clones and CTCs and exert irreplaceable regulatory effects on ferroptosis, mitochondrial homeostasis and distant metastasis; (3) atypical novel lactyltransferases and delactylases including HBO1 and SIRT5, which function independent of canonical p300/CBP and SIRT1–3, serving as brand‐new undruggable targets for precise Kla intervention. These unreported Kla readers and specific modification sites provide innovative druggable candidates beyond traditional research hotspots, and offer new directions for subsequent basic research and novel anti‐drug resistance drug development A comprehensive evaluation of druggability for these Kla‐related core therapeutic targets is summarized in Table 4.

**TABLE 4:** Comprehensive evaluation of druggability for lysine lactylation (Kla)‐related core therapeutic targets.

Target category	Specific target	Research status	Key druggability limitations	Core solution strategies
Lactate metabolism enzymes	LDHA	X‐866 (phase II); preclinical synergism with ICB	Metabolic acidosis; tumour metabolic plasticity; poor solid tumour penetration	Allosteric inhibitors + metabolic pathway inhibitors; nanoparticle targeted delivery
	MCT1/4	AZD3965 (phase I/II); no MCT4‐specific inhibitor	Retinal toxicity; MCT4 redundancy; limited tumour penetration	Dual MCT1/4 inhibitors; combination with anti‐angiogenic agents
	HIF‐1α	PX‐478 (phase I); low single‐agent efficacy	HIF‐2α compensation; poor bioavailability	Dual HIF‐1α/2α inhibitors; tumour‐hypoxia responsive prodrugs
Lysine lactylation (Kla) modifying enzymes (writer)	p300/CBP	A485 (preclinical); CBPD‐409 (preclinical PROTAC)	Poor solid tumour penetration; off‐target epigenetic dysregulation	Lysine lactylation (Kla)‐specific allosteric inhibitors; tumour‐guided PROTAC degraders
	KAT2A/ACSS2 complex	Preclinical only	Tissue redundancy; low nuclear targeting	Nuclear‐localised inhibitors; peptide‐based interaction blockers
Lysine lactylation (Kla) modifying enzymes (eraser)	SIRT2	Preclinical activators; synergism with paclitaxel	Context‐dependent pro‐tumour function; poor bioavailability	SIRT2‐selective activators; biomarker‐guided stratification
	SIRT5	Preclinical only; tissue‐specific functional heterogeneity	Low lysine lactylation (Kla) catalytic efficiency	SIRT5 lysine lactylation (Kla)‐specific activators; combination with epigenetic modulators
	HDAC1–3	Vorinostat (clinical repurposing); non‐selective inhibition	Off‐target toxicities; no lysine lactylation (Kla)‐preferential inhibitor HDAC1–3 selective inhibitors; low‐dose combination	HDAC1–3 selective inhibitors; low‐dose combination regimens
Lysine lactylation (Kla)‐related signalling targets	AKT1 (K189la)	Preclinical inhibitors; reverses trastuzumab resistance	AKT isoform redundancy; off‐target signalling inhibition	Site‐specific peptide inhibitors; PROTAC degraders for lactylated AKT1
	TFAM (K88la)	Preclinical knockdown restores EGFR‐TKI sensitivity	Mitochondrial targeting difficulty; normal tissue off‐target effects	Mitochondrial‐penetrating peptide conjugates; combination with OXPHOS inhibitors

Kla‐targeted interventions mainly (Figure 5) include suppressing lactate metabolism enzymes, regulating Kla modification enzymes and implementing combined regimens. These strategies aim to reverse Kla‐driven drug resistance by blocking lactate production, adjusting writer–eraser activities and synergising with chemotherapy or immunotherapy, thus providing a feasible translational approach to overcome tumour therapeutic resistance. Solid arrows represent activation of signalling pathways or therapeutic effects, while blunt‐ended arrows indicate inhibitory regulation of molecular targets and biological processes.

## 6 CURRENT CHALLENGES AND FUTURE DIRECTIONS

First, the dynamic regulatory network governing Kla remains highly intricate. Admittedly, existing studies and most previous reviews have largely focused on linearly summarising and enumerating recent findings published in *Nature*, *Cell*, *Cell Metabolism* and other top journals from 2023 to 2025, while lacking in‐depth integration of conflicting mechanistic conclusions and innovative hypothesis construction, which leads to simple literature repetition rather than systematic theoretical refinement. A typical unresolved paradox is the dual, contradictory role of SIRT2 in tumourigenesis and drug resistance: existing studies report that SIRT2 acts as a tumour‐suppressive delactylase in colorectal, gastric and breast cancers by removing histone Kla marks (H3K18la/H3K9la), restoring tumour suppressor gene expression and blocking glycolytic feedback loops; nevertheless, SIRT2 also exhibits oncogenic functions in liver, prostate and pancreatic cancers by preferentially modifying non‐histone substrates rather than histones, stabilising oncogenic signalling and accelerating therapeutic resistance. To resolve this mechanistic conflict, this review innovatively proposes a tissue‐metabolic substrate‐dependent dual‐function hypothesis for SIRT2: the paradoxical role of SIRT2 is determined by tumour tissue type, intratumoural lactate gradient and substrate switching between histone and non‐histone proteins. In hypoxic tumour cores with excessive lactate accumulation, SIRT2 is broadly inhibited and predominantly functions as a tumour suppressor; in oxygen‐peripheral regions and specific cancer types with moderate lactate levels, SIRT2 preferentially targets non‐histone proteins to exert pro‐tumour effects. Beyond SIRT2, other core Kla regulators including HDAC1–3, p300/CBP and AARS1/AARS2 also display context‐dependent functional discrepancies across different malignancies. This review further integrates these widespread mechanism conflicts and clarifies that intratumoural spatial heterogeneity, clonal divergence and tumour metabolic background collectively determine the substrate selectivity and downstream output of Kla modifiers, rather than simply attributing functional differences to cancer types alone, which provides a unified explanatory framework for reconciliating contradictory published results. To fill the current research gap, this review innovatively proposes a verifiable spatial–clonal evolutionary model of Kla: driven by tumour hypoxic gradient and therapeutic pressure, Kla presents progressive hyperactivation from tumour periphery to hypoxic core; during tumour metastasis and clonal evolution, CTCs undergo Kla reprogramming to form a unique modification signature distinct from primary tumours, while drug‐resistant clones gradually solidify stable inheritable Kla characteristics under long‐term drug screening, forming a complete ‘tumour core–periphery–CTCs–drug‐resistant clone’ evolutionary chain. Fluctuating lactate concentrations can dynamically influence the activity of enzymes such as p300/CBP.^156^ However, quantitative models describing the feedback loops between lactate‐generating molecules (e.g., LDHA, MCT1) and modifying enzymes (e.g., p300, SIRT2) remain poorly established. The most critical and understudied bottleneck lies in the lack of systematic investigation and quantitative profiling of intratumoural spatial and clonal heterogeneity of Kla^92^, ^170^: the lactate concentration gradient across distinct regions of the TME (e.g., hypoxic core vs. peripheral areas) drives dynamic changes in Kla modification sites, levels and substrate specificities (e.g., global histone Kla in the core vs. selective non‐histone Kla in the periphery),^1^, ^5^, ^209^ but there is a lack of high‐resolution, in situ single‐cell lactylome profiling technologies to characterise the Kla profile of each tumour cell population in situ^52^, ^220^; the Kla status of CTCs is distinct from that of primary tumour cells, but the molecular mechanisms underlying the Kla reprogramming of CTCs during EMT and circulation are not yet elucidated; drug‐resistant clones have unique Kla signatures that distinguish them from drug‐sensitive clones, but the identification of clonal‐specific Kla markers and the evolutionary dynamics of Kla signatures during therapeutic pressure are still in the exploratory stage.^221^ The lactate concentration gradient across distinct regions of the TME (e.g., hypoxic core vs. peripheral areas) drives dynamic changes in modification sites (e.g., H3K18la, H3K9la). Yet, effective techniques for in situ real‐time monitoring are currently lacking. Significant research gaps also persist in the field of non‐histone Kla. Recent studies have identified Kla on over 200 non‐histone proteins, but the regulatory networks governing their functions, particularly in processes like DNA damage repair (e.g., Kla of PARP1, which promotes chemoresistance) are not yet fully elucidated. Notably, the non‐histone Kla profiles of different tumour cell populations (core, periphery, CTCs, drug‐resistant clones) exhibit significant differences,^50^, ^58^, ^170^ but the functional specificity of non‐histone Kla in each cell population and its role in mediating cell population‐specific drug resistance and metastasis are still unclear,^24^, ^222^ which is a key scientific gap limiting the development of precise Kla‐targeted therapy.^58^, ^223^, ^224^

Notably, for core lactyltransferase targets such as p300/CBP, which have relatively sufficient preclinical data in haematological malignancies, the key bottlenecks in solid tumour application remain unresolved^183^, ^192^: (1) poor solid tumour penetration: the dense stromal barrier (e.g., collagen deposition in pancreatic cancer) and acidic TME significantly reduce the intracellular concentration of p300/CBP inhibitors (e.g., A485)^186^, ^225^; (2) lack of targeted delivery systems: non‐selective inhibition of p300/CBP in normal cells leads to global epigenetic dysregulation (e.g., haematopoietic suppression), and there is no mature tumour‐specific delivery strategy for solid tumours (e.g., EGFR/PSMA‐guided PROTAC degraders are still in preclinical development)^226^, ^227^; (3) Kla‐acetylation selectivity: current p300/CBP inhibitors simultaneously block acetylation and Kla modification, resulting in severe off‐target effects, and Kla‐binding domain‐specific allosteric inhibitors have not yet been developed.^58^, ^170^ These bottlenecks are further exacerbated by the intratumoural spatial and clonal heterogeneity of Kla: the same Kla‐targeted drug exhibits different efficacy and toxicity in different tumour cell populations, and the lack of cell population‐specific delivery systems makes it impossible to achieve precise inhibition of Kla in the tumour core, drug‐resistant clones and CTCs, while avoiding off‐target effects on normal cells and the tumour periphery with relatively normal Kla levels.^50^, ^92^ Emerging evidence (2021–2025) links non‐histone Kla to apoptotic regulation across multiple diseases: in BRAF V600mutant melanoma, lactate‐induced Kla of LSD1 at K503 stabilises its interaction with FosL1, suppressing TFRC‐mediated iron death (a ferroptotic pathway closely intertwined with apoptotic signalling) and sustaining drug‐resistant cell survival.^221^ In endometrial carcinoma, histone Kla up‐regulates USP39, which activates the PI3K/AKT/HIF‐1α pathway to inhibit apoptosis and promote malignant progression – targeting Kla with 2‐deoxy‐d‐glucose restores apoptotic sensitivity.^228^ Beyond cancer, Kla of TF Snail1 in myocardial infarction promotes endothelial‐to‐mesenchymal transition (EndMT) via TGF‐β/Smad2 signalling, exacerbating cardiac fibrosis and impairing myocardial repair by suppressing endothelial cell apoptosis.^229^ These findings expand Kla's apoptotic regulatory scope from tumours to cardiovascular diseases, highlighting the need for disease‐specific mechanistic dissection.

Furthermore, critical differences exist in the molecular mechanisms of Kla across distinct cancer types. Consequently, future basic research should focus on several key areas. First, establish a standardised quantitative quantification system for intratumoural Kla heterogeneity based on single‐cell lactylomics and spatial transcriptomics, construct computational algorithms to calculate Kla heterogeneity index and define heterogeneity threshold; second, clarify the dose‐effect relationship between Kla heterogeneity threshold and chemotherapy, targeted therapy and immunotherapy response, reveal the critical threshold value determining tumour sensitivity or drug resistance; third, take the spatial–clonal Kla evolutionary model as the core, trace the dynamic changes of Kla signature during tumour clonal evolution and treatment adaptation. It should advance novel technologies (e.g., single‐cell multi‐omics dynamic integration technology) and innovate organoid models. It should also reconstruct theoretical frameworks (such as proposing a ‘threshold effect’ theory for Kla) to define cancer‐type‐specific modification thresholds and guide precision interventions. Most importantly, future basic research must prioritise the systematic study of the intratumoural spatial and clonal heterogeneity of Kla, with three core research directions: (1) develop high‐resolution, in situ single‐cell lactylome sequencing technologies and Kla site‐specific imaging probes to characterise the Kla levels, modification sites, substrate specificities and functional outputs of the tumour core, periphery, CTCs and drug‐resistant clones at the single‐cell level, and construct a ‘tumour Kla heterogeneity atlas’ for different cancer types^52^, ^149^; (2) elucidate the molecular mechanisms underlying the Kla reprogramming of CTCs and drug‐resistant clones, including the regulatory factors driving Kla signature changes, the relationship between Kla reprogramming and EMT, stemness maintenance and drug resistance evolution, and identify CTC‐specific and drug‐resistant clone‐specific Kla markers with diagnostic and therapeutic value^1^, ^230^; (3) establish quantitative dynamic models of Kla in different tumour cell populations, describing the changes of Kla profiles in the tumour core, periphery, CTCs and drug‐resistant clones under therapeutic pressure (e.g., chemotherapy, immunotherapy, Kla inhibition), and predicting the adaptive resistance of different cell populations to Kla‐targeted therapy.^2^, ^231^ For solid tumour‐specific druggability optimisation, the following directions should be prioritised: (1) develop TME‐responsive delivery systems: construct liposomal/nanoparticle carriers modified with tumour stroma‐penetrating peptides (e.g., iRGD) to improve the penetration of p300/CBP, SIRT2 and other target modulators in solid tumours with dense stroma (e.g., pancreatic cancer, lung cancer)^232^, ^233^; (2) design Kla‐selective modulators: screen allosteric inhibitors targeting the lactyl‐CoA binding domain of p300/CBP to avoid interference with acetylation modification in normal cells and reduce off‐target toxicity^58^, ^234^; (3) establish solid tumour‐specific Kla target evaluation models: construct patient‐derived organoid (PDO) models of solid tumours with high Kla levels to evaluate the efficacy and safety of target modulators, making up for the lack of solid tumour research data in current preclinical studies.^57^ Additionally, exploring the biological functions of Kla as a tool for tumour adaptive evolution is essential. Extending beyond cancer, the context‐dependent roles of Kla in apoptotic regulation necessitate cross‐disease investigations. For instance, in neurodegenerative diseases (e.g., Parkinson's disease), lactate accumulation has been shown to induce Kla of α‐synuclein, thereby facilitating its aggregation and subsequent neuronal apoptosis.^222^ In sepsis, elevated lactate levels have been demonstrated to drive histone H3 lysine 18 Kla (H3K18la)‐mediated up‐regulation of METTL3, which exacerbates ferroptosis and apoptotic damage in alveolar epithelial cells.^177^ Future studies should also leverage single‐cell lactylomics to resolve cell‐type‐specific apoptotic regulatory networks (e.g., tumour cells vs. tumour‐infiltrating lymphocytes) and develop PDO models recapitulating Kla‐driven apoptotic resistance.^19^

Moreover, bottlenecks in drug development and clinical translation arise from concerns regarding the specificity and safety of Kla‐targeting agents. For example, p300/CBP inhibitors (e.g., A485) may disrupt normal histone acetylation in healthy cells, causing global dysregulation of gene expression (e.g., haematopoietic suppression observed in preclinical models).^184^ Similarly, SIRT2 activators could paradoxically promote tumour metastasis in certain tissues (e.g., TM activating the NF‐κB pathway in breast cancer).^36^ These specificity and safety issues are closely related to the neglect of intratumoural Kla heterogeneity in current drug development: most Kla‐targeted drugs are developed based on the global Kla profile of tumour cells, without considering the distinct Kla characteristics of different tumour cell populations and normal cells, leading to non‐specific inhibition/activation of Kla in normal cells and non‐target tumour cell populations, and thus off‐target effects and toxic side effects.^1^, ^58^ For the above problems, the core solution is to break through the technical bottleneck of solid tumour targeted delivery and Kla modification selectivity: on the one hand, utilise PROTAC technology to construct tumour‐specific degraders (e.g., CBPD‐409 modified with prostate cancer‐specific PSMA ligand) to realise the selective degradation of p300/CBP in tumour cells^227^, ^233^; on the other hand, through high‐throughput screening and molecular docking technology, develop modulators targeting the Kla‐specific functional domain of targets (e.g., SIRT2 Kla deacetylase domain activators), avoiding the non‐specific regulation of the whole protein function.^235^, ^236^ Potential solutions for these issues may involve allosteric modulation strategies. These strategies could design inhibitors specifically targeting p300's Kla‐binding domain while sparing its acetylation domain. Alternatively, optimising PROTAC degraders by exploiting tumour‐specific membrane proteins (e.g., using EGFR ligands to construct bifunctional degraders) could mitigate systemic toxicity.^237^ Recent breakthroughs offer promising directions: LSD1 inhibitors (e.g., ORY‐1001) selectively target Kla‐stabilised LSD1 in BRAFi/MEKi‐resistant melanoma, activating iron death and synergising with PD‐1 inhibitors to reverse apoptotic resistance without significant off‐target effects.^221^ Additionally, allosteric modulators targeting the lactate‐binding pocket of p300 (e.g., CCS1477) have entered phase I trials, sparing normal cell acetylation while suppressing BCL‐XL Kla and restoring apoptosis in CRC. For cardiovascular diseases, MCT1 inhibitors (e.g., CHC) reduce Snail1 Kla and EndMT, alleviating post‐infarction cardiac fibrosis by normalising endothelial cell apoptotic balance.^229^ The development of lactylome‐based liquid biopsy and in situ imaging technologies is also a key solution to the specificity and safety issues^238^: detect the Kla profiles of different tumour cell populations (core, periphery, CTCs, drug‐resistant clones) in individual patients through lactylome liquid biopsy (e.g., circulating lactylated proteins, CTC Kla markers) and in situ imaging, and conduct precise patient stratification and therapeutic regimen matching^172^, ^239^; monitor the changes of Kla levels in different tumour cell populations and normal cells during treatment in real time, and adjust the drug dose and combination regimen in a timely manner to minimise off‐target effects and toxic side effects.^52^, ^240^ The development of lactylome‐based liquid biopsies (e.g., circulating lactylated LSD1/USP39) also enables patient stratification, ensuring targeted therapies are administered to individuals with Kla‐driven apoptotic dysregulation.^236^

In addition, the research on the Kla reader proteins is still in the initial stage, the number of identified readers is limited, and their substrate binding specificity, tissue expression characteristics and regulatory mechanisms in different tumour types are not yet fully elucidated.^241^ More importantly, the Kla reader proteins exhibit distinct cell population‐specific expression and binding preferences in the tumour core, periphery, CTCs and drug‐resistant clones (e.g., TRIM33 as the dominant reader in the core, DNMT3A in drug‐resistant clones, BRD4 in CTCs),^42^, ^242^ but the functional characteristics and regulatory networks of reader proteins in each tumour cell population are completely unclear; the lack of specific reader targeting tools also restricts the in‐depth exploration of Kla downstream functional output in different cell populations, which is an important scientific gap to be filled in the field of Kla research.^92^ The lack of specific reader targeting tools also restricts the in‐depth exploration of Kla downstream functional output, which is an important scientific gap to be filled in the field of Kla research.^44^ Future research on Kla reader proteins must focus on cell population specificity: identify the dominant reader proteins of different tumour cell populations (core, periphery, CTCs, drug‐resistant clones) through single‐cell lactylome and pull‐down experiments, elucidate their substrate binding specificity and downstream regulatory networks in mediating cell population‐specific drug resistance and metastasis,^241^ and develop reader protein‐specific inhibitors and binding blockers (e.g., TRIM33 inhibitors for the tumour core, DNMT3A blockers for drug‐resistant clones, BRD4 inhibitors for CTCs) to target the Kla downstream functional output in different tumour cell populations.^38^ Additionally, explore the synergistic regulation of writer–eraser–reader proteins in each tumour cell population and develop multi‐target combination drugs targeting the entire Kla regulatory network of specific cell populations.^243^ Future research should prioritise the systematic study of the intratumoural spatial and clonal heterogeneity of Kla and strengthen the identification and functional verification of unreported Kla reader molecules, novel tumour‐specific Kla modification sites and atypical lactylation‐modifying enzymes beyond traditional hot targets such as LDHA, MCT1/4, p300/CBP and SIRT2, so as to excavate more innovative precise therapeutic targets for overcoming tumour drug resistance.

In summary, regarding the cooperative regulation between Kla and other PTMs, fundamental research and clinical practice aimed at overcoming tumour drug resistance should focus on specific objectives. They should elucidate crosstalk networks (e.g., Kla‐acetylation competition, Kla‐phosphorylation cascades^196^). They should pioneer the development of dual‐targeting epigenetic drugs. They should also devise novel therapeutic strategies to dynamically reprogram modification landscapes. Most critically, all research and development of Kla‐targeted therapies must take the intratumoural spatial and clonal heterogeneity of Kla as the core premise^14^: from the basic research of Kla molecular mechanisms to the design of targeted drugs, from the development of combination therapies to the clinical translation and personalised application,^48^, ^199^ fully consider the distinct Kla profiles and functional characteristics of the tumour core, periphery, CTCs and drug‐resistant clones,^52^ and construct a complete research and development system^16^ These efforts will reposition Kla from an ‘accomplice in drug resistance’ to a ‘therapeutic target’, ultimately overcoming the formidable barrier in solid tumour treatment. Beyond tumour drug resistance, integrating Kla's apoptotic regulatory roles into broader disease contexts – such as targeting LSD1 Kla for neurodegenerative diseases or Snail1 Kla for cardiovascular fibrosis – opens new therapeutic frontiers.^19^, ^222^, ^229^ Future directions must also address PTM crosstalk in apoptotic pathways: for instance, Kla of LSD1 antagonises its phosphorylation (by ERK1/2) to sustain anti‐apoptotic signalling, while SIRT5‐mediated delactylation enhances BCL‐XL ubiquitination and degradation.^194^, ^228^ Developing dual‐target agents (e.g., LSD1/p300 inhibitors) that disrupt Kla‐PTM crosstalk could achieve synergistic apoptotic induction across diseases. Finally, advancing real‐time, cell population‐specific Kla monitoring technologies (e.g., FRET‐based probes targeting H3K18la/Snail1la, CTC Kla flow cytometry detection)^244^, ^245^ and lactylome‐based liquid biopsy technologies will enable dynamic therapeutic response assessment and personalised regimen adjustment,^48^ accelerating the clinical translation of Kla‐targeted therapies that consider intratumoural heterogeneity.

## 7 CONCLUSION

Tumour drug resistance remains a central bottleneck restricting long‐term efficacy of cancer therapy, and metabolic–epigenetic crosstalk has emerged as a critical regulatory axis underlying treatment failure. As a newly identified PTM directly driven by the Warburg effect, Kla links metabolic reprogramming, epigenetic remodelling, DNA repair, immune escape and apoptotic suppression into a cohesive network that drives tumour drug resistance. This review systematically delineates the molecular basis, regulatory mechanisms, heterogeneous characteristics and therapeutic potential of Kla in drug‐resistant tumours, providing a unified conceptual framework for understanding metabolism‐controlled therapeutic resistance.

The core innovations of this review lie in three aspects:

First, we clarify Kla as a reversible metabolic–epigenetic switch governed by the writer–eraser–reader machinery, which dynamically modulates histone and non‐histone substrates to reshape transcriptional programs and protein function.
Second, we define intratumoural spatial and clonal heterogeneity as a key feature of Kla‐driven resistance: hypoxic tumour cores display global hyper‐Kla and intrinsic resistance; peripheral regions exhibit inducible Kla and acquired resistance; CTCs harbour unique Kla signatures for survival and metastasis; and drug‐resistant clones maintain stable Kla patterns that distinguish them from sensitive populations.
Third, we integrate cancer‐type‐specific Kla mechanisms and reveal that Kla promotes resistance via four conserved pathways: enhanced DNA damage repair, epigenetic reprogramming of resistance genes, immunosuppressive TME remodelling and disrupted survival–apoptosis balance.

From a clinical implication perspective, this review establishes Kla as a promising actionable target for overcoming MDR. Targeting lactate metabolism, lactyltransferases, delactylases or downstream effectors can resensitise tumours to chemotherapy, targeted therapy and immunotherapy. Notably, therapeutic strategies must account for Kla heterogeneity: combinatorial regimens tailored to tumour core, periphery, CTCs and resistant clones will achieve more durable responses than single‐target approaches. Kla‐related signatures also hold potential as predictive biomarkers for drug response and patient stratification.

Despite rapid progress, critical challenges remain, including incomplete characterisation of Kla readers, lack of modification‐selective inhibitors and difficulty in targeted delivery across heterogeneous tumours. Future studies should focus on single‐cell lactylomics, tumour‐type‐specific Kla networks and clinically feasible targeted agents.

In summary, Kla represents a fundamental metabolic–epigenetic driver of tumour drug resistance with profound biological and clinical relevance. Decoding Kla‐mediated mechanisms will not only deepen our understanding of tumour adaptive strategies but also foster the development of novel precision therapies to conquer drug resistance and improve patient prognosis. This review establishes a clear and verifiable spatial–clonal evolutionary model of Kla among tumour core, periphery, CTCs and drug‐resistant clones, and innovatively proposes two forward‐looking research directions: the quantitative technical system of Kla intratumoural heterogeneity and the predictive value of Kla heterogeneity threshold for clinical therapeutic response, which makes up for the lack of systematic theoretical model and prospective academic perspective in current researches, and provides new research ideas and theoretical support for the follow‐up in‐depth exploration of Kla heterogeneity and precise targeted therapy.

## AUTHOR CONTRIBUTIONS

Drafted the manuscript and designed the figures: Juan Li, Yan Shang and Hailong Zhao. Substantially contributed to analysis and manuscript preparation: Juan Li, Yan Shang, Maiqun Zuo, Haojie Wu, Shenglong Yang and Hailong Zhao. Helped perform the analysis and engaged in constructive discussions: Hailong Zhao. Conceived and critically revised the manuscript and figures: Juan Li, Yan Shang, Maiqun Zuo, Haojie Wu, Shenglong Yang and Hailong Zhao. Wrote the paper: Juan Li, Yan Shang, Maiqun Zuo, Haojie Wu, Shenglong Yang and Hailong Zhao. All authors have read and agreed to the published version of the manuscript.

## FUNDING INFORMATION

This study was funded by the National Natural Science Foundation of China Regional Project (Grant No.: 82060503), Guizhou Provincial Basic Research Program (Natural Science) (Grant No. QianKeHeJiChu‐MS [2026] 900), Guizhou Province Science and Technology Plan Project (Grant Nos.: QKHJC‐ZK [2022] General 622) and National College Students’ Innovation and Entrepreneurship Training Program (S202310661178, S202310661167, S2024106612300).

## CONFLICT OF INTEREST STATEMENT

The authors declare no conflicts of interest.

## Data Availability

The data referenced in this study were mainly derived from the publicly available database PubMed. No new raw datasets were generated in this work, and all supporting data are fully presented within the article.
